# Impacts of leachates from livestock carcass burial and manure heap sites on groundwater geochemistry and microbial community structure

**DOI:** 10.1371/journal.pone.0182579

**Published:** 2017-08-03

**Authors:** Man Jae Kwon, Seong-Taek Yun, Baknoon Ham, Jeong-Ho Lee, Jun-Seop Oh, Weon-Wha Jheong

**Affiliations:** 1 Korea Institute of Science and Technology, Gangneung, Republic of Korea; 2 KU-KIST Green School, Korea University, Seoul, Republic of Korea; 3 Department of Earth and Environmental Sciences, Korea University, Seoul, Republic of Korea; 4 National Institute of Environmental Research, Incheon, Republic of Korea; Universita degli Studi di Milano-Bicocca, ITALY

## Abstract

We investigated the impacts of leachates from a swine carcass burial site and a cow manure heap on the geochemical and microbiological properties of agricultural water samples, including leachate, groundwater from monitoring wells and background wells, and stream water. The leachate from the livestock burial site showed extremely high electrical conductivity, turbidity, and major ion concentrations, but low redox potential and dissolved oxygen levels. The groundwater in the monitoring wells adjacent to both sites showed severe contamination from the leachate, as indicated by the increases in EC, turbidity, Cl^-^, and SO_4_^2-^. Bacteria from the phylum Firmicutes and Bacteriodetes and Archaea from the phylum Euryarchaeota were the major phyla in both the leachates and manure heap. However, the class- or genus-level components of these phyla differed markedly between the leachate and manure heap samples. The relative abundance of Firmicutes decreased from 35% to 0.3~13.9% in the monitoring wells and background wells at both sites. The Firmicutes in these wells was unlikely to have originated from the transportation of leachate to the surrounding environment because Firmicutes genera differed drastically between the leachate and monitoring wells. Meanwhile, sulfate-reducing bacteria (SRB) from the livestock carcass burial site were detected in the monitoring wells close to the leachate. This was likely because the release of carcass decomposition products, such as organic acids, to adjacent areas improved the suitability of the local environments for SRB, which were not abundant in the leachate. This study highlights the need to better understand microbial community dynamics along groundwater flow paths to evaluate bacterial transport in subsurface environments and provides new insights into the effective management of groundwater quality at both farm and regional scales.

## Introduction

Surface water and groundwater contamination by livestock-waste-derived solutes and microorganisms and its risks to human health have long been recognized [1]. Leachates released from both mass carcass burial sites and livestock fecal waste heaps can have negative impacts on groundwater quality, and are a major public concern [2–4]. These leachates are a potential source of both conventional contaminants (e.g., chemical oxygen demand, total organic carbon, total nitrogen, total phosphorus, and solids) and biologically active contaminants (e.g., pathogens, antimicrobials and steroid hormones) that can move through subsurface environments [5].

Since 2010, major outbreaks of foot-and-mouth disease (FMD) have occurred in South Korea and a total of 3.4 million head of livestock, including 1.2 million swine, were buried in 2010–2011 [6]. Dead animals must be disposed safely to prevent issues such as odor, pathogens, and excess nutrients [7]. However, high levels of microbial contaminants, including bacteria and nitrogenous compounds, have been detected in groundwater near livestock carcass burial sites in South Korea [8, 9]. Therefore, monitoring microorganisms and groundwater quality near burial sites is essential to ensure the safety of the local public.

There has been increased concern worldwide over the effects of microbial components from livestock carcass burial and fecal waste management on groundwater quality [1, 8]. For example, pathogens and solutes presented in fecal waste can move to surrounding environments and may result in water and soil contamination. Hutchison et al (2004) reported that both fresh and stored livestock manures contained substantial proportion (8–22%) of pathogens such as *E*. *coli* O157:H7, *Salmonella*, and *Campylobacter* [10]. On the other hand, a previous report showed that complete animal decomposition could require more than 2 years and could generate greenhouse gases and leachates containing high levels of chemical contaminants [11, 12]. Thus, long-term leachate release may act as a continuous source of contaminants, providing growth substrates for soil microorganisms in subsurface environments [9]. The subsurface microorganisms present near livestock carcass burial sites can be classified as enteric microorganisms directly from livestock carcasses, carcass-decomposing microorganisms, or indigenous soil microorganisms [9].

To prevent groundwater contamination by harmful pathogens in the future, it is critical to track microbial sources and evolution precisely and to address animal carcasses and manure as a source [13]. However, it is difficult to identify representative microorganisms in subsurface environments using conventional culturing methods. Several studies have investigated the microbial communities of livestock burial and waste disposal sites using culture-dependent analyses [14, 15]. These studies provided evidence of the possible presence of specific microorganisms, including pathogens and indigenous soil microorganisms. However, using culture-dependent methods to detect soil microorganisms may give limited information (i.e., false-positive and false-negative results can occur) about the microorganisms present in and near livestock burial and fecal waste disposal sites. A recent study demonstrated that the composition of cultured isolates (i.e., isolation using specific growth media) selected both a small and unrepresentative share of soil microorganisms compared to that sampled using a culture-independent method, 454-pyrosequencing of total soil DNA [16].

Several studies have investigated the compositions of and changes in microbial communities in leachates from livestock carcass burial sites [17–19], but little or no effort has been expended to determine the spatial distribution of microbial taxa coupled with geochemical dynamics at livestock carcass sites. In addition, although microbial communities originating from intestinal tracts may be similar regardless of livestock species, no or little study has attempted to compare the structures of microbial communities found at livestock-derived contamination sites such as swine burial sites with those at cow manure heap sites.

Therefore, we investigated the evolution of microbial community composition and groundwater chemistry influenced by leachates from swine carcass burial and cow manure heaps along groundwater flow paths. The microbial community compositions were evaluated using culture-independent Illumina MiSeq high-throughput sequencing. The objectives of this study were to 1) understand how the leachates from livestock-derived contamination sites impact the geochemical and microbiological properties of subsurface environments; 2) identify which microbial communities are predominant in swine carcass burial site and cow manure heap sites; 3) determine which factors control the microbial community compositions around these sites, and 4) examine to what spatial distance microorganisms in leachates can transport to surrounding subsurface environments. The results of this study will improve our understanding of the transport of bacteria from livestock burial sites and manure heaps to subsurface environments and provide insights into the effective management of groundwater quality and microbial contamination.

## Materials and methods

### Study area and sampling

**Fig 1:**
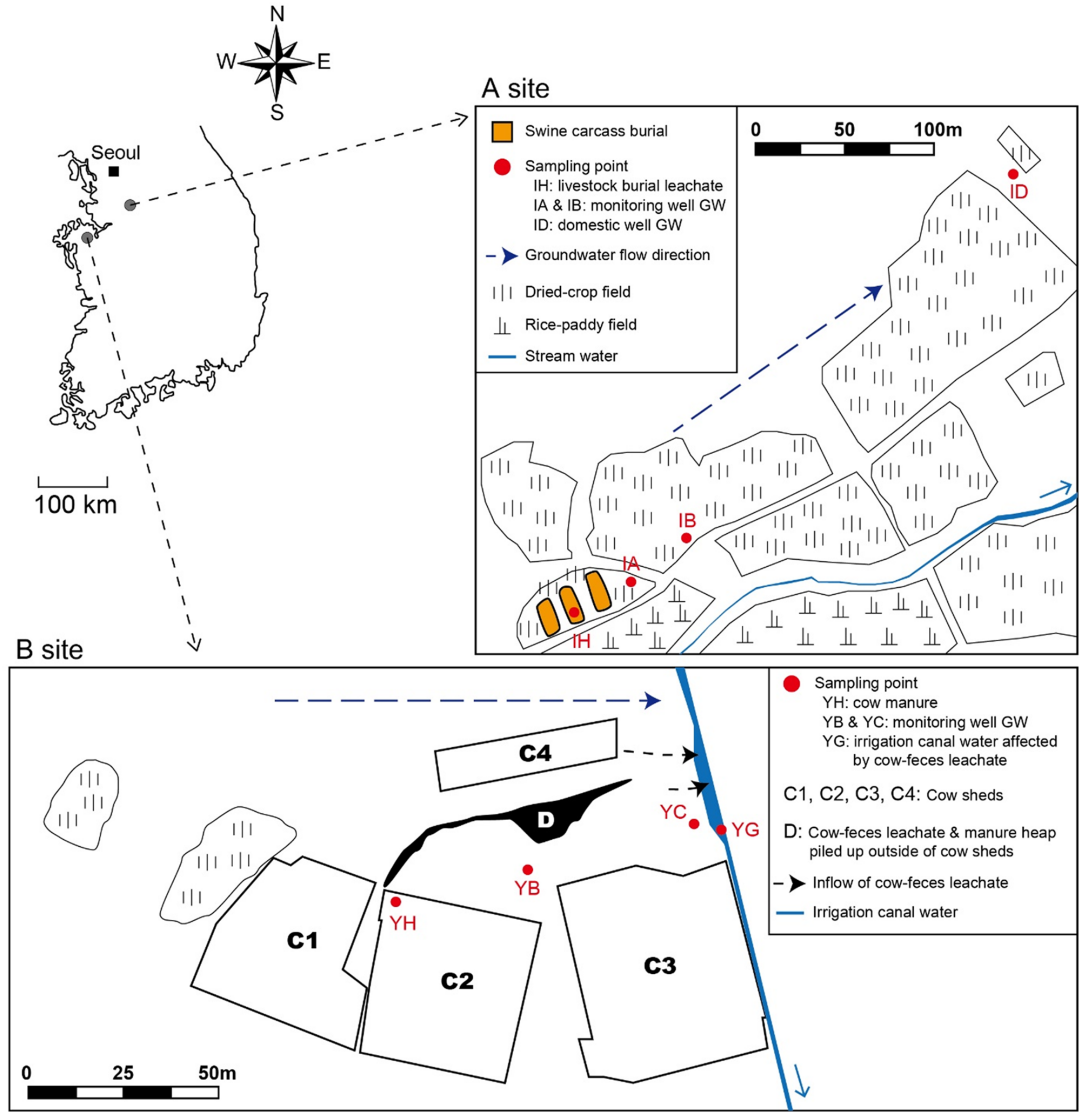
Study areas: livestock carcass burial site (A) and livestock manure heap site (B). The locations of the livestock burial sites, multi-level monitoring wells (IA and IB), and domestic groundwater wells (ID) are marked on the detailed map of the survey area. The locations of cow sheds, multi-level monitoring wells (YB and YC), the manure sampling point (YH), and the surface water stream runoff sampling point (YG) are marked on a detailed map of the survey area. The groundwater flow direction is represented by a blue arrow.

To answer the objectives of our study, we selected two field sites influenced by livestock-derived contaminants. The swine carcass burial site (A site) and cow manure heap site (B site) examined in this study were located in the central and western regions of the Republic of Korea, respectively (Fig 1A and 1B). The cow manure heap (‘D’ in Fig 1B) was located outside cow sheds on a farm with 140 dairy cows, and was not a government-run waste disposal site. In January 2011, three livestock carcass burial events occurred due to an FMD outbreak in the area, resulting in the burial of 3,989 swine. Both sites were surrounded predominantly by dryland or paddy fields. The underlying aquifer systems were formed from unconsolidated colluvial materials deposited at the base of hill slopes after weathering from Jurassic granite bedrock. The hydraulic conductivities of the aquifers at the livestock burial site and livestock manure heap differed greatly according to the sediment particle size distribution. The hydraulic conductivities at the two locations were estimated at 0.33 and 0.14 m d^-1^, respectively.

To evaluate the relationships between groundwater quality and microbial community compositions and to determine the microbial transport from the contaminant sources to surrounding environments, water samples were collected along the groundwater flow direction near the carcass burial site and manure heap, including leachate, groundwater from monitoring and background wells, and surface stream water (Fig 1). Multi-level monitoring wells (sampling sites: IA, IB, YB, and YC) with several sampling depths (IA: −5, −10, −17 m; IB: −10 m; YB: −6 m; YC: −9, −15 m) were installed at both study sites. Installation of the monitoring wells was performed in 2012 (A site) and in 2013 (B site) using a rotary drilling device. Borehole drilling was undertaken to the depth of weathered bedrock (Jurassic granite or Precambrian gneiss) underlying colluvial or alluvial sediments. The bundle of multi-level groundwater samplers [consisting of polyvinyl chloride (PVC) pipes (2.5 cm diameter) and polyethylene tubes (0.5 cm diameter) with the bottom parts slotted (15 cm long) and wrapped with stainless steel screen for water sampling] at different depths was placed into the borehole and then the monitoring wells were backfilled with excavated materials, sand and bentonite. After the completion of well installation, the monitoring wells were immediately purged by a peristaltic pump to remove mixed stagnant water. The location and sampling depth of the monitoring wells were determined by considering the general groundwater flow direction and location of the pollution sources. In other words, the monitoring wells are located downstream (very close to the pollution sources) of the carcass burials (A site) and cow manure heaps (B site) based on the groundwater flow directions in the study sites (Fig 1).

For water sampling, each well was pumped continuously at 0.5 L min^-1^ using a peristaltic pump (7523–30 Masterflex; Cole Palmer Vernon Hills, IL, USA). The samples were collected after clearing stagnant water from the wells. Well purging prior to groundwater sampling was done to remove stagnant water that may not be representative of *in-situ* groundwater quality. For this, the monitoring wells were purged approximately three times volume (electrical conductivity (EC) value also becomes constant) of water stored in the wells to avoid water quality disturbance by excessive purging. The leachate sample in the carcass burials was collected by inserting the sampling tube into the pipe which connected to the inside of the burials from the roof, and then collected the leachate by using the peristaltic pump with slow pumping rate. Between 28 October and 4 November 2013, 11 samples (eight groundwater, one surface stream, one carcass leachate, and one feces leachate) were collected. Several potentially unstable parameters, including temperature, redox potential (Eh), pH, EC, and dissolved oxygen (DO), were measured at the field sites.

The water samples were collected in capped bottles connected directly to sampling tubes and the peristaltic pump to minimize air contact. The samples were filtered through a 0.45-μm membrane filter into pre-cleaned 60-mL polyethylene bottles. For the cation analysis, samples were preserved by adding concentrated HNO_3_ to keep the pH below 2. All sample bottles were filled completely and capped with elastic laboratory film to avoid air contact, and then stored at 4°C until the analysis.

For the microbial community analysis, biomass was collected by filtering the retentate from 1–5 L of water samples through a 47-mm filtration unit (Nalgene Reusable Filter Holder; Thermo Fisher Scientific Korea, Seoul, Republic of Korea) fitted with a 0.2-μm filter (Pall Gelman Laboratory, Ann Arbor, Michigan, USA). The filters were preserved in a 50-mL sterilized conical tube on dry ice until they could be stored at −20°C in the lab. Fecal waste (YH in Fig 1B) was collected using a sterilized spatula and preserved in a tube at −20°C until the experiments were completed.

### Analytical methods

The temperature, pH, EC, Eh, and DO of all water samples were measured using Orion portable meters (Orion 5-star RDO Multiparameter Meters; Thermo Scientific, Beverly, MA, USA). The portable meters were calibrated and checked before the measurements. Alkalinity was determined in the field via volumetric titration using 0.05 N HNO_3_ [20].

Cations were analyzed by with inductively coupled plasma-atomic emission spectroscopy (Optima 3000XL; Perkin-Elmer, Waltham, MA, USA) and anions were analyzed with ion chromatograph (DX-120; Dionex, Sunnyvale, CA, USA) at the Center for Mineral Resource Research, Korea University, South Korea. Turbidity was determined using a turbidity meter (HI93703 Portable Turbidity Meter; Hanna, Woonsocket, RI, USA). Total viable colony counts were obtained by plating diluted groundwater samples on a solid agar medium according to a method described previously [21].

### Microbial community analysis

#### DNA extraction

Approximately one-fourth of a filter paper or 0.5 g of fecal waste was dispensed into a sterile micro-centrifuge tube. Total genomic DNA was extracted using an i-genomic Soil DNA Extraction Mini Kit (iNtRON, Seongnam, South Korea) with a bead-beating disruption apparatus according to the manufacturer’s directions. The DNA concentration was quantified using a Qubit fluorometer (Invitrogen, USA) following manufacturer’s instructions.

#### 16S rRNA gene library preparation

The V4 regions of the 16S rRNA gene were amplified using F515 (5ʹ-GTGCCAGCMGCCGCGGTAA-3ʹ) and R806 (5ʹ-GGACTACVSGGGTATCTAAT-3ʹ) (Bates et al., 2011). The thermocycler conditions for the PCR amplification were 95°C for 3 min, followed by 25 cycles of 95°C for 30 s, 55°C for 30 s, and 72°C for 30 s, followed by a final extension at 72°C for 5 min and holding at 4°C. The amplicons were purified using AMPure XP beads (Beckman Coulter, Brea, CA, USA). Nextera XT indexes (Illumina) were added at half reaction to remove short library fragments, including index 1 (i7) and index 2 (i5), from the population. Then, 1 μL (1/50 dilution volume) of the final product was used to verify the size of the DNA, confirming the expected size of 300–350 bp. The DNA was quantified with the KAPA Library Quantification Kit for Illumina platforms (KAPA Biosystems, Wilmington, MA, USA) or Qubit fluorometer (Invitrogen, USA) according to the manufacturer’s instructions. Paired-end (2 × 254 bp) sequencing was performed at Macrogen Inc. (Seoul, Republic of Korea) using a MiSeq^™^ platform (Illumina, San Diego, USA).

#### Sequencing data analysis

The total sequencing count was 24,017, and the sequencing depth-to-average count per sample was 2,183 (S1 Table). The sequencing data were analyzed using QIIME ver.1.8.0 [22]. Sequences were clustered into operational taxonomic units at 97% similarity with the UCLUST algorithm [23]. To calculate species diversity and richness within individual samples, alpha diversity analyses (e.g., Chao1, ACE, and Shannon Index) for the statistical tests were processed using the QIIME script. To measure similarity among communities, beta diversity was analyzed, and two- and three-dimensional principle coordinate analysis (PCoA) plots were constructed. Non-metric multidimensional scaling (NMDS) analyses were also performed with the package vegan implemented in R [24], based on the microbial community information (relative abundance of each OTU detected by either MiSeq sequencing or clone library). The vector fitting of environmental variables to the NMDS ordination was determined using the vegan package with 16 major components of physical and chemical characteristics. Significance was determined based on Bray–Curtis distances and 10,000 random permutations.

## Results and discussion

### Water quality changes with distance from the contaminant source and depth

**Fig 2:**
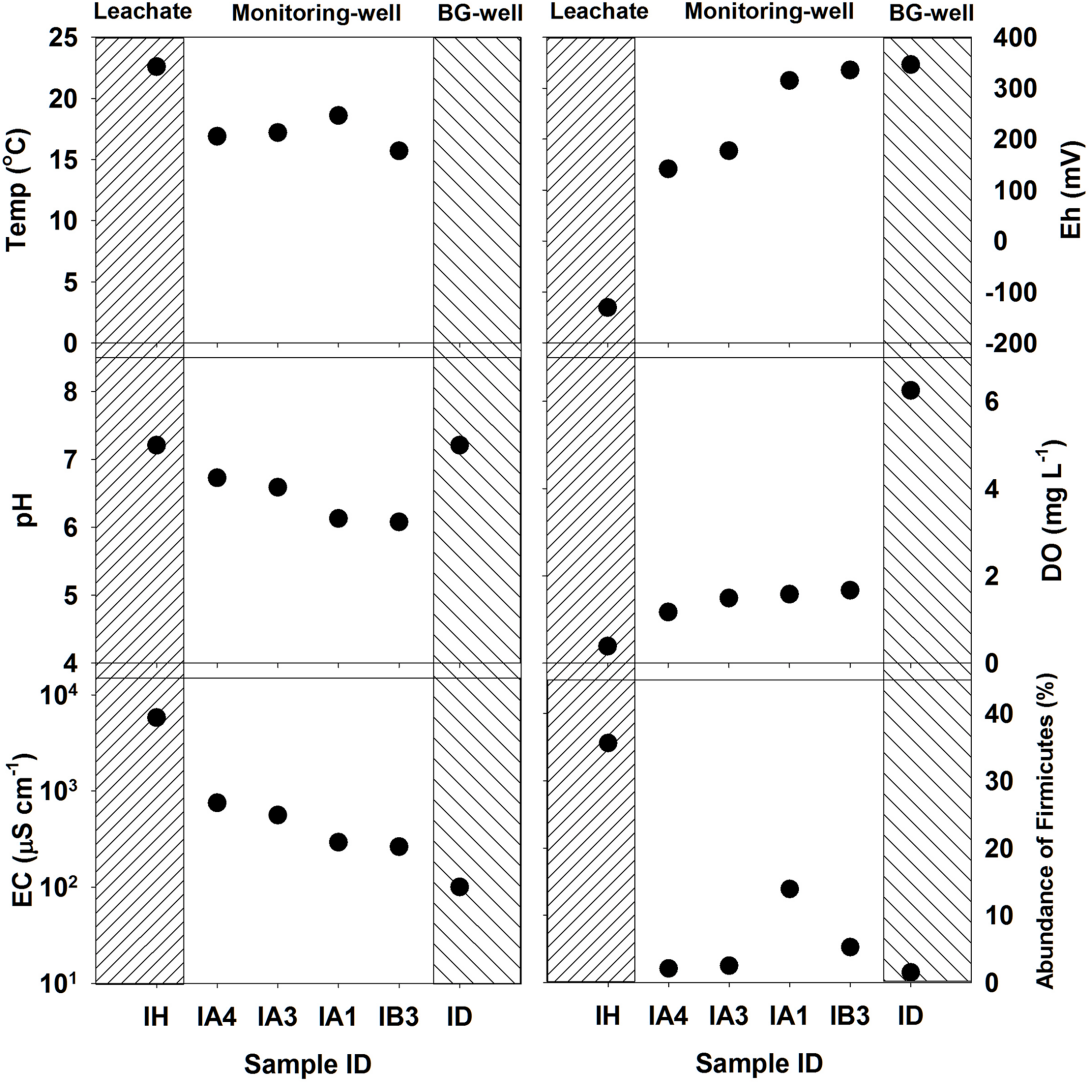
Variations in physicochemical properties and Firmicutes abundance of samples with increasing distance from the contaminant sources.

The leachate (IH) from the livestock carcass burial site showed extremely high values of EC (~5,780 μS cm^-1^), turbidity (4,650 NTU), and major ion concentrations, but low Eh (−131 mV) and DO (0.4 mg L^-1^) compared to the background groundwater (ID) values of EC (100 μS cm^-1^), turbidity (0.1 NTU), Eh (346 mV), and DO (6.3 mg L^-1^) (Table 1 and Fig 2). These results suggested that the microbial decomposition of carcass biomass caused decreases in DO and Eh in groundwater. Furthermore, the carcass leachate had the highest ion concentrations, particularly Cl^-^ (1,121 mg L^-1^), SO_4_^2-^ (1,828 mg L^-1^), NO_3_^-^ (1,965 mg L^-1^), and HCO_3_^-^ (5,340 mg L^-1^). These ions in the leachate likely originated from carcass decomposition, as discussed below. As the water sampling distance increased from the contamination source (i.e., leachate, sample IH), EC, turbidity, Cl^-^, SO_4_^2-^, NO_3_^-^, and HCO_3_^-^ decreased remarkably, whereas Eh and DO increased. In particular, Cl^-^ concentrations decreased rapidly but continuously with increasing distance from the leachate source, and showed the lowest concentration in the background well. Chloride ion is a well-known tracer of groundwater and contaminant flow because it is relatively inert and is not biodegradable [25]. Therefore, our hydrochemical observations suggested that the leachate released from the swine burial site was highly diluted with ambient groundwater upon flow. Table 1 shows the results of the water quality analysis around the cow manure heap. Unlike the carcass burial site, the manure heap did not exhibit clear trends in DO, EC, and Eh levels with distance from the contaminant source (‘D’ in Fig 1B), likely because there were multiple manure sources around the farm. However, the surface water (i.e., runoff) sample (YG) results showed some evidence of contamination, with relatively high NO_3_^-^, SO_4_^2-^, and Cl^-^ concentrations. Moreover, the groundwater chemistry around the cow sheds (YB and YC) also indicated substantial contamination with NO_3_^-^, SO_4_^2-^, and/or Cl^-^, which were much higher than those concentrations around the carcass burial site.

**Table 1:** Summary of the physical, chemical, and biological properties of the samples.

Sampling site		Livestock carcass burial site						Livestock manure heap site				
Sample ID		IH	IA4	IA3	IA1	IB3	ID	YH	YG	YB1	YC2	YC3
Description		Leachate	GW (M)^1^	GW (M)	GW (M)	GW (M)	GW (B)^2^	Feces	SW^3^	GW (M)	GW (M)	GW (M)
**Distance from contaminant source (m)**		0	10	10	10	55	311	-	46*	14*	39*	39*
**Water table (mbgl)**^4^		0	3.50	3.50	3.50	3.47	3.43	ND^11^	0	4.95	6.18	6.18
**Sampling depth (m)**		3	17	10	5	10	45	ND	0	6	9	15
**On-site analysis**	**Temp. (°C)**	22.6	16.9	17.2	18.6	15.7	n.a.	ND	15.1	17.2	19.2	18.4
	**pH**	7.2	6.7	6.6	6.1	6.1	7.2	ND	9.5	5.9	5.7	6.3
	**Eh (mV)**^5^	-131	142	177	315	336	346	ND	282	375	464	379
	**EC** **(μS cm**^**-1**^**)**^6^	5780	753	561	292	264	100	ND	512	291	505	650
	**DO** **(mg L**^**-1**^**)**^7^	0.4	1.2	1.5	1.6	1.7	6.3	ND	11.1	5.1	5.9	5.1
	**Turbidity (NTU)**^8^	4650	2.42	2.32	0.44	1.53	0.09	ND	1.36	0.30	0.26	0.17
**Total Colony Count** **(CFU mL**^**-1**^**)**^9^		1757	23.7	15.7	33.3	0.3	0	ND	4900	7.7	2	7.7
**Dissolved conc. (mg L**^**-1**^**)**	**Ca**^**2+**^	106.9	21.6	21.0	23.6	23.5	10.7	ND	37.5	13.1	25.0	26.1
	**Mg**^**2+**^	137.1	7.4	6.3	5.4	4.4	1.2	ND	8.6	4.6	12.1	12.9
	**Na**^**+**^	52.6	15.3	14.2	13.1	12.7	9.0	ND	35.8	25.1	50.5	70.5
	**K**^**+**^	167.6	25.2	16.2	3.7	6.1	0.4	ND	9.6	4.3	4.1	17.9
	**SiO**_**2**_	27.0	18.5	17.8	18.6	18.0	33.4	ND	4.0	38.6	17.0	11.8
	**Cl**^**-**^	1121.3	19.0	20.6	11.6	9.2	2.1	ND	115.1	20.2	58.1	89.7
	**SO**_**4**_^**2-**^	1827.9	18.3	31.6	27.6	2.0	1.2	ND	197.5	1.0	106.8	27.0
	**NO**_**3**_^**-**^	1965.2	1.5	2.2	24.9	nd	10.5	ND	156.9	61.2	9.5	58.4
	**CO**_**3**_^**2-**^	nd	nd	nd	nd	nd	nd	ND	1.9	nd	nd	nd
	**HCO**_**3**_^**-**^	5340.0	86.9	249.4	96.1	97.6	59.5	ND	59.5	44.2	44.2	115.9
	**F**^**-**^	nd^10^	0.56	nd	0.10	0.12	0.97	ND	0.74	0.09	nd	nd

^1^ GW (M) = groundwater from monitoring well.

^2^ GW (B) = groundwater from background location.

^3^ SW = surface water (canal water).

^4^ mbgl = meters below ground level.

^5^ Eh = redox potential.

^6^ EC = electrical conductivity.

^7^ DO = dissolved oxygen.

^8^ NTU = nephelometric turbidity units.

^9^ the drinking water guideline of total colony count in South Korea is <100 CFU mL^-1^.

^10^ nd = not detected.

^11^ ND = not determined.

* distance from ‘D’ in Fig 1B.

The plot of the groundwater samples in the Piper diagram showed the presence of several water types (S1 Fig). The groundwater types of samples IH (leachate) and IA4 were CO_3_ type with carbonate hardness >50%, suggesting that the addition of CaO for viral disinfection at the carcass burial site increased HCO_3_^-^ concentrations [26]. The groundwater types of samples IB3 and ID were Ca–Cl type, while those of samples YB1 and YC3 were Na–K–Cl type.

Vertical variations in the physical and chemical properties were investigated using the multi-level monitoring wells (wells IA and YC). Well IA showed decreases in temperature, Eh, DO, and Firmicutes abundance and increases in pH and EC with increasing depth (Fig 2). The physical and chemical properties of the groundwater at depths of 5, 10, and 17 m suggested that the plume of leachates most strongly affected the groundwater of IA at a depth of 17 m. Interestingly, NO_3_^-^ concentrations in IA decreased remarkably between depths of 10 and 17 m (24.9 to 1.5 mg L^-1^), suggestive of NO_3_^-^ reduction via microbial nitrate reduction [27] and simple dilution with the surrounding groundwater. This observation was supported by an increase in and predominance of nitrate-reducing microorganisms in this microbial community (see *Sulfurimonas* in section 3.3). In the case of YC, the on-site measured parameters, including DO (5.1–5.9 mg L^-1^) and EC (505–650 μS cm^-1^), were relatively similar at both measured depths (9 and 15 m), but more Na^+^, Cl^-^, NO_3_^-^, and HCO_3_^-^ and less SO_4_^2-^ were observed at the depth of 15 m (Table 1). We cannot reasonably explain these differences because samples were collected at only two depths, but it is likely that the hydrogeochemical and geological conditions at this site are vertically heterogeneous.

### Comparison of microbial community compositions between the carcass burial and manure heap sites

Amplicon libraries of all samples were constructed to characterize the bacterial and archaeal communities in the carcass leachate, groundwater, manure, and surface water, and to determine community development along the groundwater flow path. To compare microbial diversity levels according to sampling location (i.e., distance of groundwater flow from the source) and between the carcass burial site and manure heap samples, rarefaction analyses were conducted with 868 randomly selected sequences per sample (S2 Fig). The steep slopes of the rarefaction curves (plots of the number of observed species as a function of the number of sequences sampled) suggested that a fraction of the species diversity in the samples remains to be discovered. However, the rarefaction curves indicated that species richness was generally higher in samples from the carcass burial site than in those from the manure heap (S2 Fig). These results suggested that the anoxic and anaerobic conditions and the leachate chemical compositions (e.g., various organic acids) at the carcass burial site might have created metabolic complexities that supported an increase in bacterial diversity [28].

**Fig 3:**
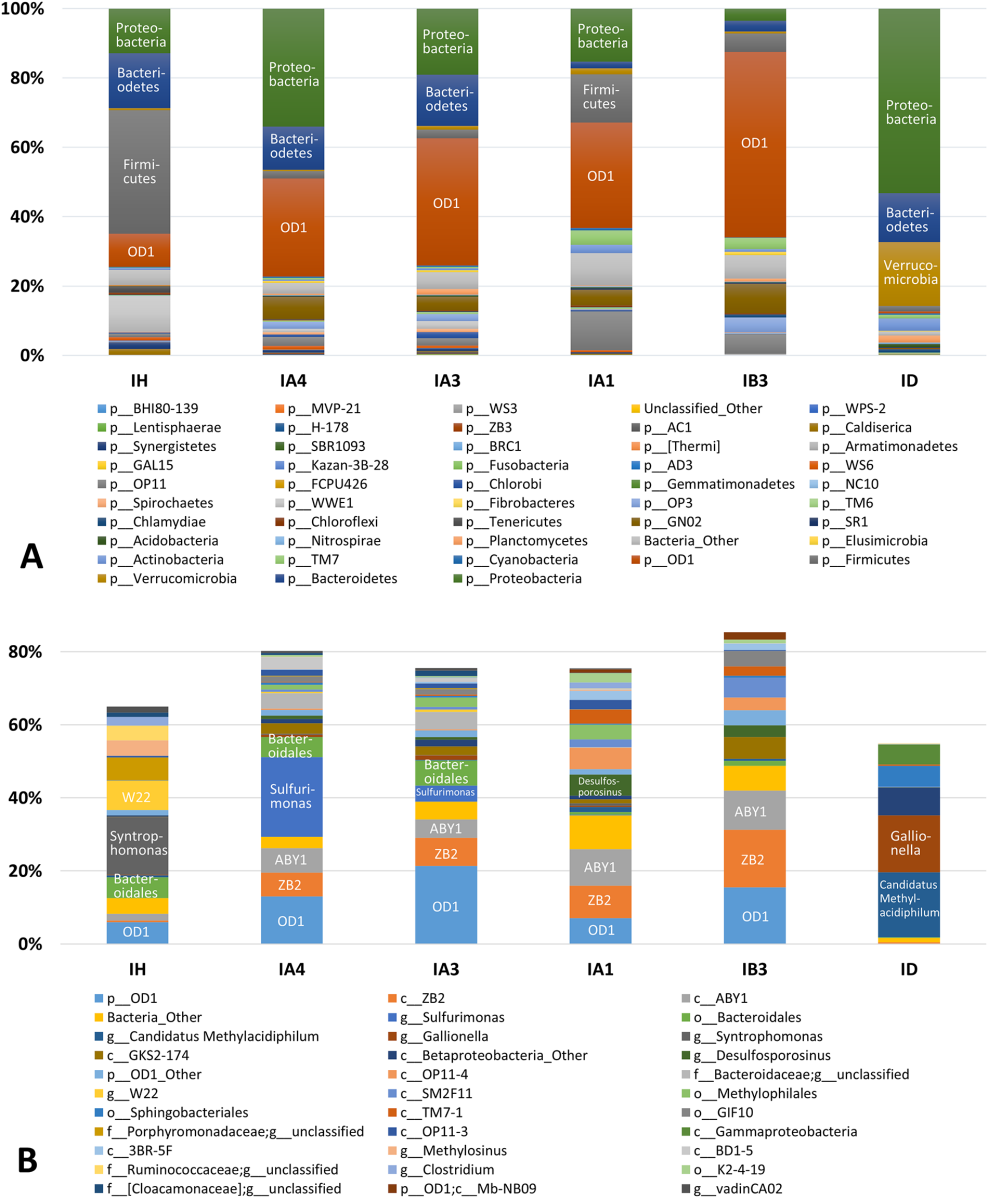
Bacterial community composition at the phylum level (A) and genus level (B) of the samples collected from the livestock carcass burial site.

**Fig 4:**
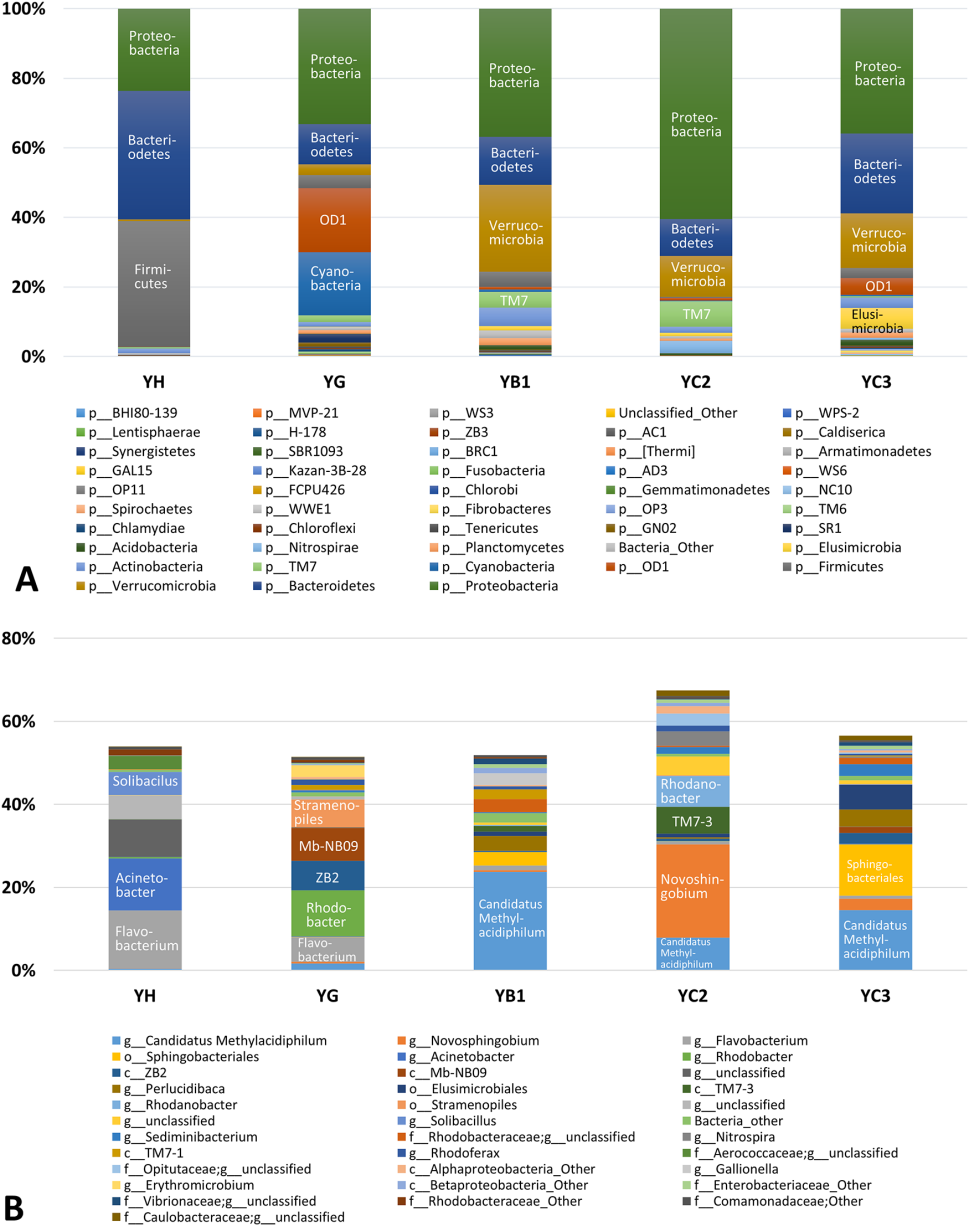
Bacterial community composition at the phylum level (A) and genus level (B) of the samples collected from the livestock manure heap site.

The relative abundances of the bacterial phyla and genera in each sample were calculated as the percentage of sequences belonging to a particular phylum to the total 16S rRNA gene sequences recovered from each sample (Figs 3 and 4, S2 and S3 Tables). Firmicutes, Bacteriodetes, and Proteobacteria were the major phyla in both the carcass leachate (IH; 35.6%, 15.9%, and 12.9%, respectively) and the manure heap (YH; 36.1%, 36.9%, and 23.7%, respectively) (Figs 3 and 4). Microbes in these phyla are commonly found in animal gastrointestinal tracts [29]. Our results were similar to those of [30], which showed that the sequences matching Firmicutes and Bacteriodetes initially predominated in leachates from swine carcasses, but shifted continuously over time. Genus-level comparisons of bacterial communities between the carcass burial site and manure heap are discussed in section 3.3.

**Fig 5:**
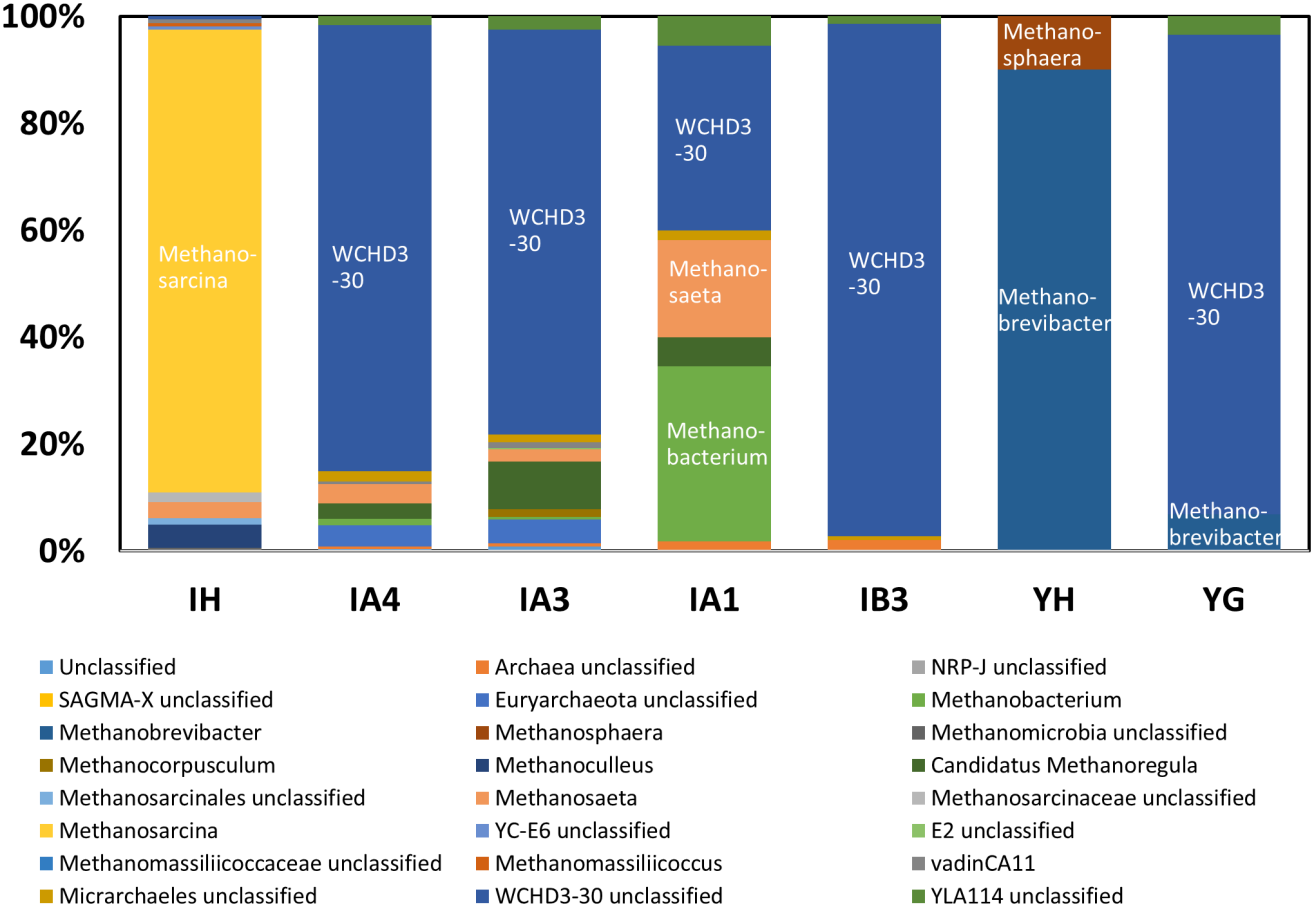
Archaeal community composition at the genus level of samples collected from the livestock carcass burial and livestock manure heap sites. The community composition data are not presented for samples with <20 sequences (i.e., ID, YB1, YC2, and YC3).

The phylum- or genus-level components of the archaeal communities are shown in Fig 5 and S4 Table. Although the primers used in this study are known to amplify both bacterial and archaeal sequences [31], there were significantly fewer sequences related to Archaea than to Bacteria (S1 Table). Only samples containing >20 archaeal sequences are discussed hereafter. Similar to the bacterial community composition, the archaeal communities differed markedly between the carcass burial site and manure heap (Fig 5). Sequences related to the genus *Methanosarcina* dominanted in the carcass leachate (IH), whereas *Methanobrevibacter* and *Methanosphaera* were the major genera in the manure heap (YH). These genera are common rumen methanogens [32], and have been detected in paddy fields [33]. However, these genera were replaced by the archaeal genera *Methanobacterium*, *Methanosaeta*, and WCHD3-30 (unclassified) in the carcass leachate site, and WCHD3-30 (unclassified) in the manure heap.

### Factors controlling microbial community distribution

Turbidity was extremely high in the leachate (IH) due to the high concentration of suspended particles, and was lower in samples IA and IB (Table 1). By comparison, the turbidity in the background groundwater was near zero. Suspended particles are an important component of microbial groundwater contamination because of the ability of bacteria to attach onto particulates. For example, the number of attached microorganisms on solid particles in an aquifer was reported to be one to two orders higher than that of free-living microorganisms [34, 35]. In addition, microbes associated with suspended particles had greater survivability [36]. In this study, the total colony counts at both sites generally increased with increasing turbidity (Table 1), suggesting that solid particles could be vectors of bacterial contamination in aquifers.

**Fig 6:**
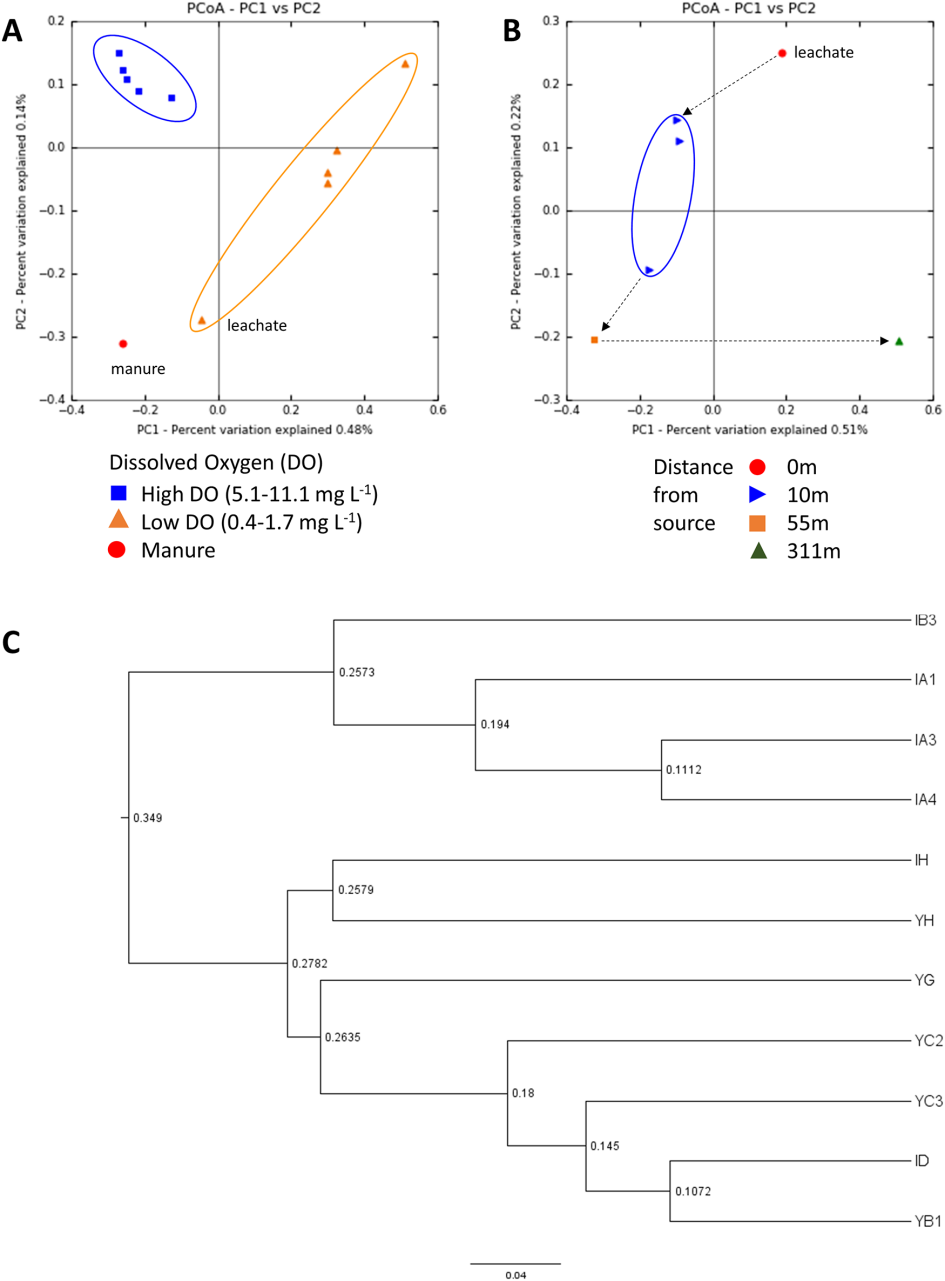
Principal coordinate analysis (PCoA) and UniFrac analysis of the bacterial communities associated with the leachate, groundwater, and feces samples. PCoA plots of the samples showing clustering by dissolved oxygen (A) and by distance from the carcass leachate (B). Unweighted pair group method with arithmetic mean (UPGMA) distance tree (C) of the MiSeq bacterial community structure. The percentages in parentheses for the PCoA indicate the proportion of variation explained by each ordination axis. The numbers on the nodes indicate the bootstrapping values for each node.

DO had a significant role in microbial community distribution. The weighted UniFrac distances were visualized in PCoA plots (Fig 6A and 6B). The results showed that the bacterial communities differed between samples with low DO (0.4–1.7 mg L^-1^) and those with high DO (5.1–11.1 mg L^-1^). For instance, the phylum OD1, also known as Parcubacteria, was identified in livestock carcass burial site and manure heap samples with low DO (except sample YG), suggesting that this phylum might proliferate in anoxic environments in the study area [37]. This implied that DO had an important role in the development of bacterial communities. Moreover, the bacterial community in the manure sample was similar to that in the sample with low DO.

The livestock contaminant source (i.e., swine carcass versus cow manure) was another critical factor controlling microbial community distribution. An unweighted pair group method with arithmetic mean (UPGMA) tree generated from the UniFrac distance matrix (Fig 6C) showed that the bacterial communities of all samples were grouped distinctly into those from the carcass burial site and those from the manure heap site. As mentioned in the previous section, the microbial communities of the carcass leachate (IH) and manure heap (YH) samples were similar at the phylum level. However, the class- or genus-level components within Firmicutes and Bacteriodetes differed markedly between the carcass leachate (IH) and manure heap (YH) samples. Notable taxa of Firmicutes included *Syntrophomonas* (15.8%), Ruminococcaceae (unclassified genus) (4.1%), and *Clostridium* (2.4%) in the carcass leachate, while *Solibacillus* (5.5%) and Aerococcaceae (unclassified genus) (3.3%) were present in the fecal waste (Figs 3 and 4 and S3 Table). In addition, Bacteriodetes taxa detected in the carcass leachate (Porphyromonadaceae [unclassified genus] [6.2%], Bacteroidales [unclassified genus] [7.3%]) differed markedly from those in the manure heap (*Flavobacterium* [13.9%], Flavobacteriaceae) [unclassified genus] [9.0%], Porphyromonadaceae [unclassified genus] [5.8%]). Interestingly, the bacterial composition differed from that in a previous study [30], in which the bacterial communities in decomposing swine carcass leachates showed a predominance of uncultured *Tissierella* spp. and *Peptostreptococcus* spp. In addition, another study [38] reported that *Clostridium*, SMB53, *Prevotella*, *Treponema*, *Ruminococcus*, *Faecalibacterium*, *Enterococcus* (class: Bacilli), *Trichococcus*, *Facklamia*, *Caryophanon*, and unclassified Lactobacillales were the predominant taxa in pig intestines. These results suggest that the microbial community composition in the leachates from the carcass burial and manure heap sites may differ significantly according to regional location, livestock type, and feed type.

The chemical compositions of leachate can be an important influencing factor of microbial community composition, as it can serve as a substrate for indigenous microbial growth. For example, sequences related to sulfate-reducing bacteria (SRB) within Firmicutes (e.g., *Desulfosporosinus*) were present in the monitoring wells close to the carcass leachate (IA and IB) (Fig 3 and S3 Table). This was likely because the release of carcass decomposition products (e.g., organic carbons such as organic acids, alcohols, and cyclic hydrocarbons, sulfur, and nitrogen compounds) [39, 40] to adjacent areas improved the suitability of local environments for SRB that were not abundant in the carcass leachate or the background wells. Sequences similar to *Sulfurimonas* (class: Epsilonproteobacteria) were only observed in well IA, and they increased in abundance with increasing depth (Fig 5 and S3 Table). *Sulfurimonas* species, which are capable of sulfur (e.g., sulfide, elemental sulfur, thiosulfate, and sulfite) oxidation coupled with oxygen or nitrate reduction, are commonly identified in sulfidic environments (e.g., hydrothermal deep-sea vents), and in marine and terrestrial sediments [41]. The decrease in DO and NO_3_^-^ concentrations and increase in the abundance of sequences related to *Sulfurimonas* at well IA with increasing depth suggest that *Sulfurimonas* is important to the redox process in such subsurface environments.

**Fig 7:**
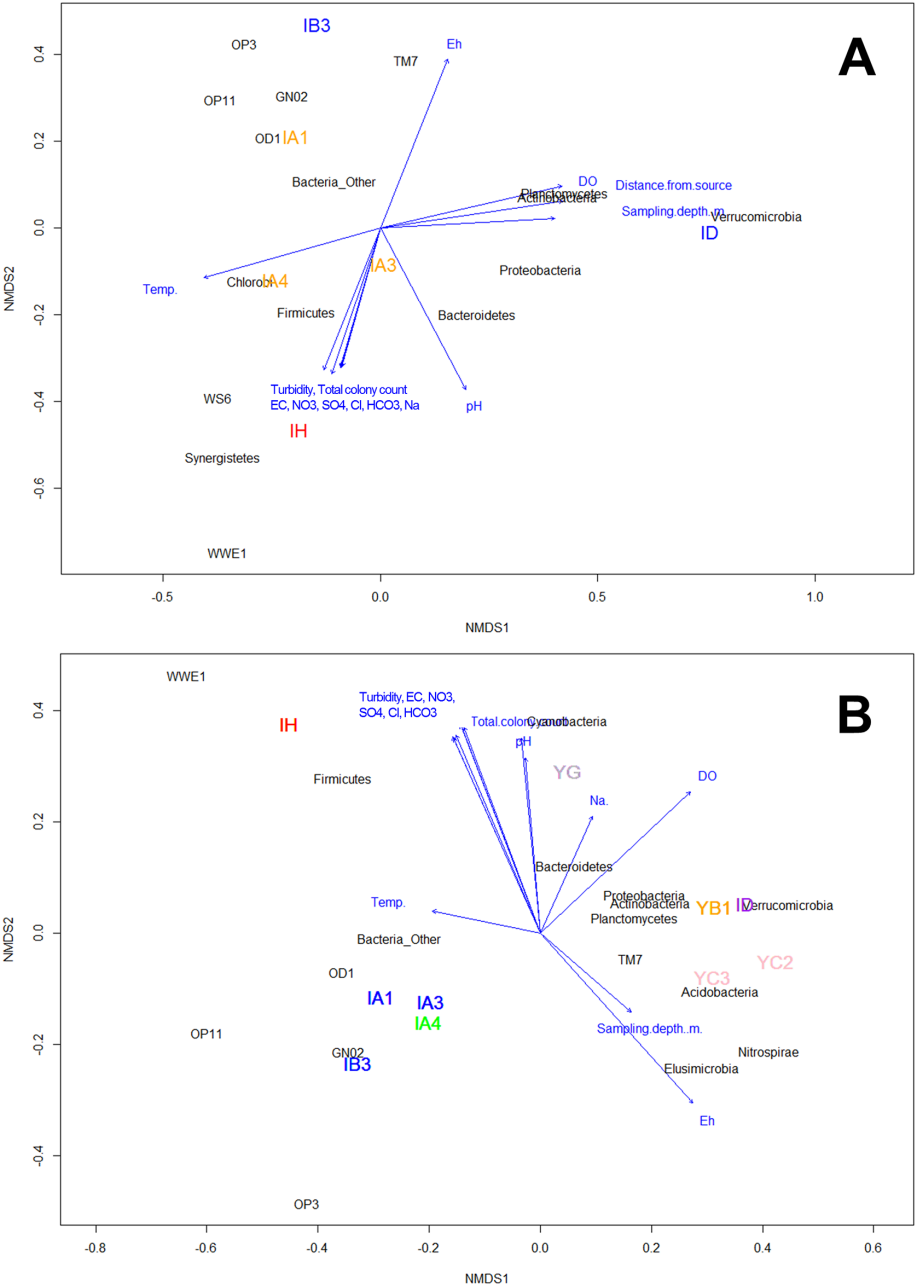
Non-metric multidimensional scaling (NMDS) plots of environmental variables and microbial community compositions at the phylum level in water samples collected from the livestock burial site (A) and both the livestock burial and manure heap sites (B). NMDS analysis within the vegan package of R software package based on dissimilarities calculated using the Bray–Curtis index of bacterial community composition for the relative abundance of each OTU in relation to the environmental variables. The direction and length of the vectors of water quality factors from Table 1 are computed by Bray–Curtis distances the "envfit()" function in the vegan package.

In a complimentary analysis, NMDS analysis using environmental variables (Table 1) and bacterial phyla (S2 Table) in the livestock burial sites were conducted to confirm whether geochemical factors affect community structure (Fig 7A). Among factors we tested, distance from source, sampling depth, temperature, pH, Eh, EC, and DO showed significant relationships with community compositions (r^2^ > 0.85, P < 0.1) (S5 Table). The microbial community in the samples close to the livestock burial sites was closely related to turbidity, total colony count, EC, and major ions, while that in the samples far the burial sites was correlated with relatively high DO and Eh. The result of NMDS using environmental variables and bacterial phyla in both sites together (Fig 7B) revealed that bacterial community compositions correlated significantly with several environmental variables including Eh, EC, DO, turbidity, and total colony count (r^2^ > 0.56, P < 0.08) and NO_3_ˉ, SO_4_^2^ ˉ, HCO_3_ˉ, and Clˉ (r^2^ > 0.68, P < 0.05) (S5 Table).

### Microbial transport potential from the carcass burial site and manure heap

Leachates from livestock carcass burial sites or livestock manure heap could cause microbial contamination of groundwater. Although the transport of bacteria from a source is dependent on various hydrogeological characteristics, including hydraulic conductivity and particle size distribution [42], microorganisms can be transported from a source to a well along a hydraulic gradient. In this study, the concentration of microorganisms (i.e., total colony counts) was positively correlated with Na^+^ and Cl^-^ concentrations and somewhat correlated with SO_4_^2-^ concentrations. In particular, the relative abundance of Firmicutes decreased abruptly in the monitoring wells and background wells (Figs 3 and 4). Therefore, it is critical to evaluate whether this phylum can be transported to adjacent areas via groundwater flow paths. The relative abundance of Firmicutes decreased from 35.6% to 2.0% over ~10 m from IH at the carcass burial site and from 36.1% to 4.4% over ~7 m from YH at the manure heap site (S2 Table). Some Firmicutes could be considered to have moved from the source areas to the surrounding wells. However, the occurrence of Firmicutes in monitoring wells was not explained well by their transport from the carcass leachate or manure heap to the surrounding environments because the genus-level classifications of Firmicutes taxa differed markedly between the leachates and monitoring wells. The bacterial and archaeal community compositions in each sample suggested that the transport of bacteria from the carcass burial site and manure heap to surrounding areas did not occur over meter-scale distances.

## Conclusions

The physical and chemical properties of the groundwater in wells near livestock carcass burial and manure heap sites were directly influenced by the leachates. However, most of the dissolved inorganic compounds were rapidly diluted by ambient groundwater. Nitrate concentrations decrease even further due to microbial denitrification. The results of the 16S rDNA analysis showed that the genus-level microbial community compositions differed markedly between the swine carcass burial and cow manure heap sites. Turbidity, DO concentration, leachate composition, and contaminant source were the major factors controlling the microbial community distribution in the study area. The results of the community analysis supported the low probability of direct microbial transport or contamination from the carcass burial sites to surrounding environments, as the sequences related to enteric bacteria found in the leachate were not detected in adjacent wells. This study suggests that the transport of microbes from livestock carcass burial and manure heap sites to surrounding areas is unlikely over meter-scale distances but that the release of leachate results in changes in geochemical conditions that can promote the growth of specific members within microbial communities, such as SRB. This study provides insights into the effective management of groundwater quality and microbial contamination at farm and regional scales.

## Supporting information

S1 Fig Piper diagram of the water type for the groundwater samples collected in this study. (JPG)

S2 Fig Rarefaction analysis of 16S rDNA sequences sampled from the groundwater at the livestock carcass burial and livestock manure heap sites. Each rarefaction curve represents the number of operational taxonomic units (OTUs; clusters of sequences with >97% similarity) detected based on the sampling intensity of the libraries.
(TIF)

S1 Table Summary of the sequence analysis. (DOCX)

S2 Table Classification and relative abundance of Bacteria (phylum level) at the livestock carcass burial and livestock manure heap sites. (DOCX)

S3 Table Classification and relative abundance of Bacteria (genus level) at the livestock carcasses burial and livestock manure heap sites. (DOCX)

S4 Table Classification and relative abundance of Archaea (genus level) at the livestock carcass burial and livestock manure heap sites. (DOCX)

S5 Table Non-metric multidimensional scaling (NMDS) data of environmental variables and microbial community compositions at the phylum level in water samples collected from the livestock burial site (A) and both the livestock burial and manure heap sites (B). (DOCX)
