# Supplementary figures and images for: Impacts of leachates from livestock carcass burial and manure heap sites on groundwater geochemistry and microbial community structure
Source: PLoS One. 2017 Aug 3;12(8):e0182579. doi: 10.1371/journal.pone.0182579 (PMC5542392; doi:10.1371/journal.pone.0182579)

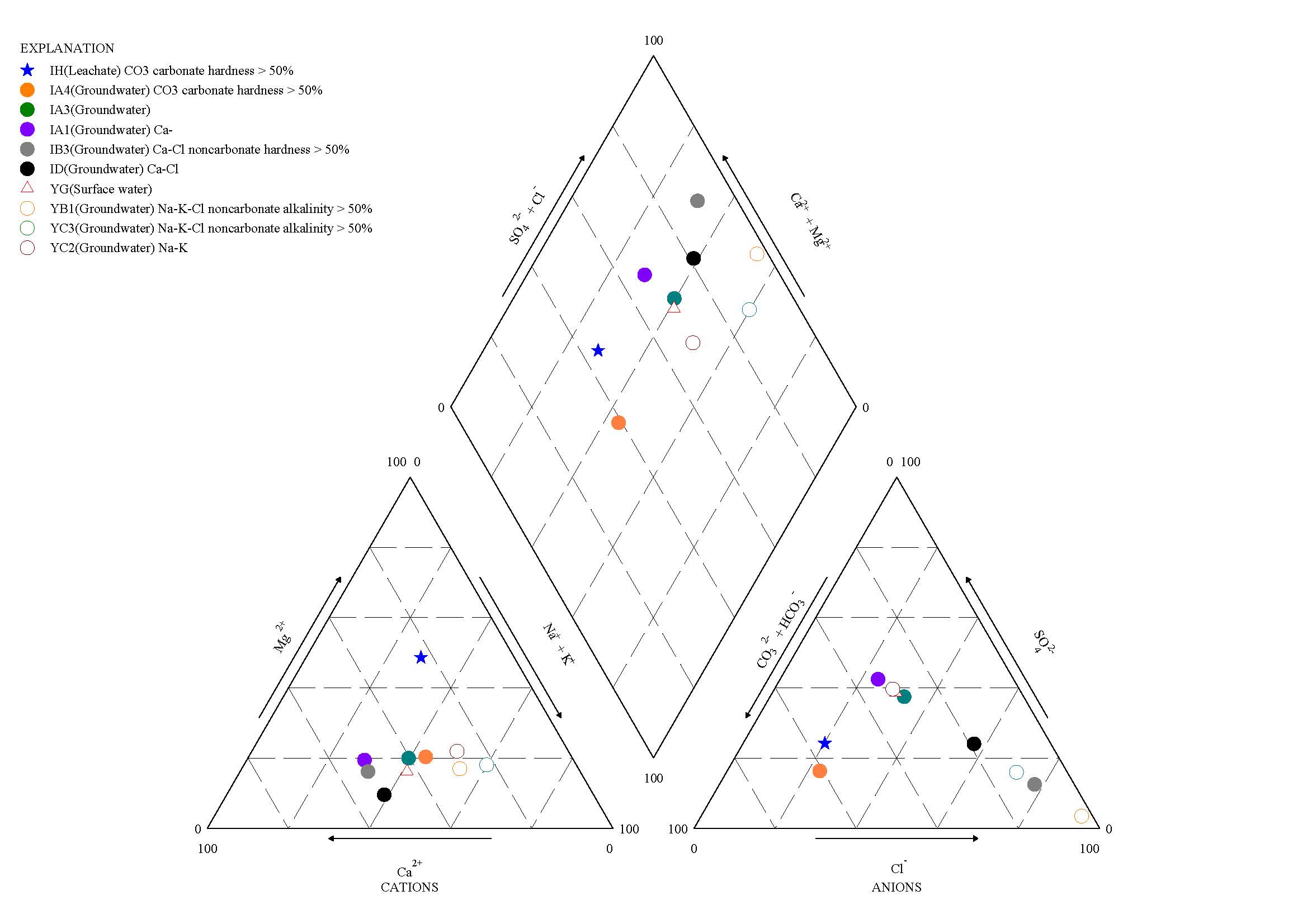

Supplement: S1 Fig — (JPG) [file pone.0182579.s001.jpg]

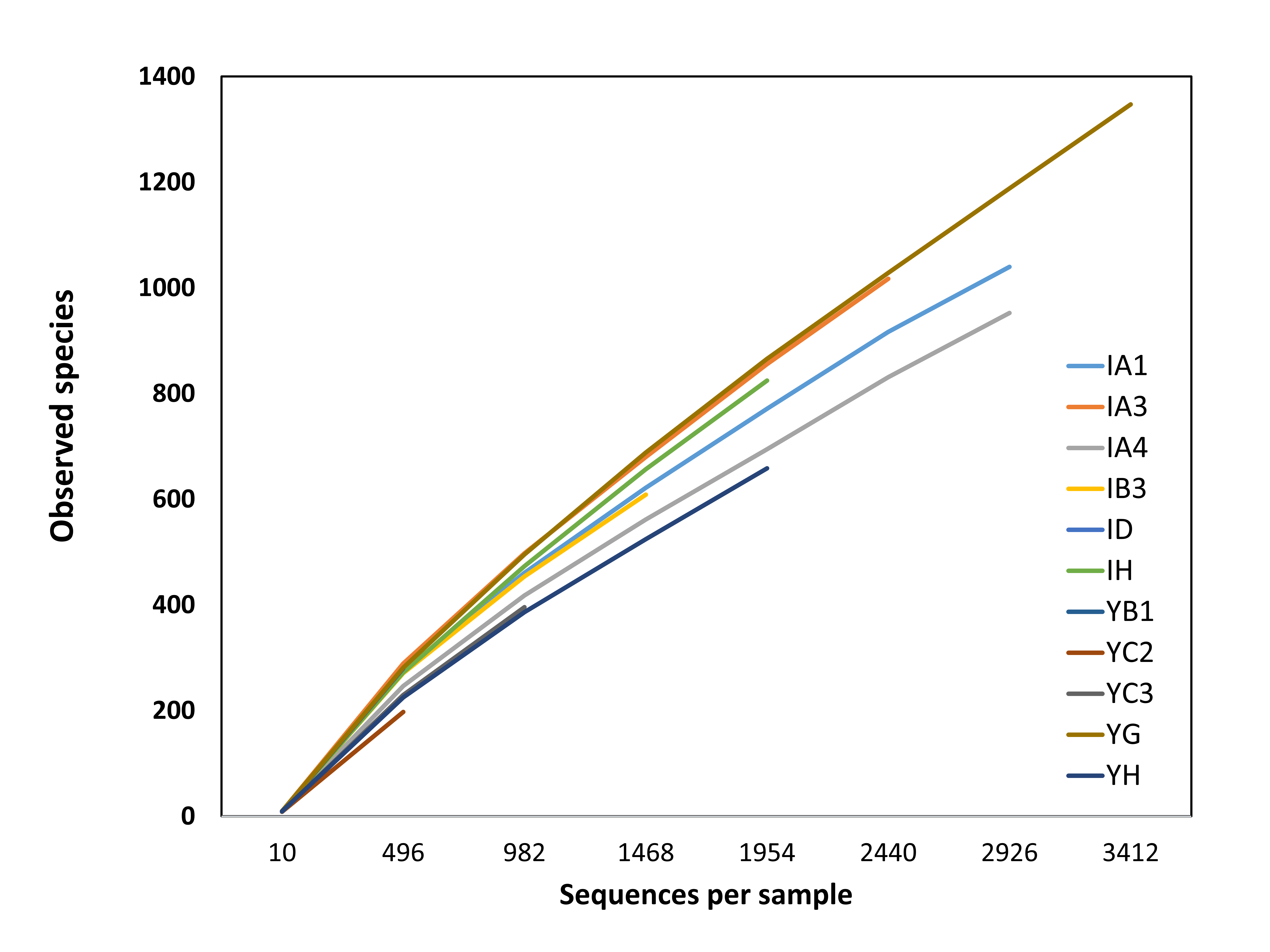

Supplement: S2 Fig — Each rarefaction curve represents the number of operational taxonomic units (OTUs; clusters of sequences with >97% similarity) detected based on the sampling intensity of the libraries. (TIF) [file pone.0182579.s002.tif]
