# Supplementary material for: Impacts of leachates from livestock carcass burial and manure heap sites on groundwater geochemistry and microbial community structure
Source: PLoS One. 2017 Aug 3;12(8):e0182579. doi: 10.1371/journal.pone.0182579 (PMC5542392; doi:10.1371/journal.pone.0182579)
Supplement: S1 Table — (DOCX) [file pone.0182579.s003.docx]

S1 Table. Summary of the sequence analysis.

| **Counts/sample summary** | | **All** | **Bacteria** | **Archaea** |
| --- | --- | --- | --- | --- |
| **Number of total samples** | | 11 | 11 | 11 |
| **Number of total observations (97% OTU)** | | 7482 | 7340 | 142 |
| **Total counts** | | 24022 | 22996 | 1026 |
| **Statistics** | **Min** | 738 | 734 | 0 |
|  | **Max** | 3443 | 3414 | 358 |
|  | **Median** | 2409 | 2389 | 29 |
|  | **Mean** | 2183 | 2091 | 93 |
|  | **Std. dev.** | 976 | 913 | 115 |
| **Livestock**  **carcass**  **burial site** | **IH** | 2554 | 2390 | 164 |
|  | **IA4** | 3404 | 3157 | 247 |
|  | **IA3** | 3161 | 2803 | 358 |
|  | **IA1** | 3075 | 3020 | 55 |
|  | **IB3** | 1759 | 1615 | 144 |
|  | **ID** | 1191 | 1189 | 2 |
| **Livestock manure**  **heap site** | **YH** | 2409 | 2389 | 20 |
|  | **YG** | 3443 | 3414 | 29 |
|  | **YB1** | 979 | 979 | 0 |
|  | **YC2** | 738 | 734 | 4 |
|  | **YC3** | 1309 | 1306 | 3 |
