# Supplementary material for: Impacts of leachates from livestock carcass burial and manure heap sites on groundwater geochemistry and microbial community structure
Source: PLoS One. 2017 Aug 3;12(8):e0182579. doi: 10.1371/journal.pone.0182579 (PMC5542392; doi:10.1371/journal.pone.0182579)
Supplement: S2 Table — (DOCX) [file pone.0182579.s004.docx]

S2 Table. Classification and relative abundance of Bacteria (phylum level) at the livestock carcass burial and livestock manure heap sites.

| **#OTU ID** | **Livestock carcass burial site** | | | | | | **Livestock manure heap site** | | | | |
| --- | --- | --- | --- | --- | --- | --- | --- | --- | --- | --- | --- |
|  | **IH** | **IA4** | **IA3** | **IA1** | **IB3** | **ID** | **YH** | **YG** | **YB1** | **YC2** | **YC3** |
| BHI80-139 | 0.00 | 0.00 | 0.00 | 0.07 | 0.00 | 0.00 | 0.00 | 0.00 | 0.00 | 0.00 | 0.00 |
| MVP-21 | 0.00 | 0.00 | 0.00 | 0.00 | 0.00 | 0.08 | 0.00 | 0.00 | 0.00 | 0.00 | 0.00 |
| WS3 | 0.00 | 0.06 | 0.11 | 0.00 | 0.00 | 0.00 | 0.00 | 0.00 | 0.00 | 0.00 | 0.00 |
| Unclassified_Other | 0.00 | 0.03 | 0.11 | 0.00 | 0.00 | 0.08 | 0.00 | 0.00 | 0.00 | 0.00 | 0.00 |
| WPS-2 | 0.00 | 0.06 | 0.00 | 0.03 | 0.00 | 0.17 | 0.00 | 0.00 | 0.00 | 0.00 | 0.00 |
| Lentisphaerae | 0.08 | 0.00 | 0.11 | 0.13 | 0.00 | 0.00 | 0.00 | 0.00 | 0.00 | 0.00 | 0.00 |
| H-178 | 0.00 | 0.16 | 0.18 | 0.00 | 0.00 | 0.00 | 0.00 | 0.00 | 0.00 | 0.00 | 0.00 |
| ZB3 | 0.00 | 0.25 | 0.21 | 0.03 | 0.00 | 0.00 | 0.00 | 0.00 | 0.00 | 0.00 | 0.00 |
| AC1 | 0.00 | 0.32 | 0.32 | 0.00 | 0.00 | 0.00 | 0.00 | 0.00 | 0.00 | 0.00 | 0.00 |
| Caldiserica | **1.76** | 0.03 | 0.29 | 0.26 | 0.00 | 0.00 | 0.00 | 0.00 | 0.00 | 0.00 | 0.00 |
| Synergistetes | **1.92** | 0.60 | 0.75 | 0.33 | 0.00 | 0.00 | 0.00 | 0.00 | 0.00 | 0.00 | 0.00 |
| SBR1093 | 0.00 | 0.00 | 0.00 | 0.00 | 0.00 | 0.00 | 0.00 | 0.03 | 0.00 | 0.00 | 0.00 |
| BRC1 | 0.00 | 0.00 | 0.00 | 0.00 | 0.00 | 0.00 | 0.04 | 0.00 | 0.00 | 0.00 | 0.00 |
| Thermi | 0.04 | 0.03 | 0.00 | 0.00 | 0.00 | 0.00 | 0.04 | 0.00 | 0.00 | 0.00 | 0.00 |
| Armatimonadetes | 0.21 | 0.06 | 0.07 | 0.07 | 0.43 | 0.00 | 0.00 | 0.06 | 0.00 | 0.00 | 0.00 |
| GAL15 | 0.00 | 0.00 | 0.00 | 0.00 | 0.00 | 0.00 | 0.00 | 0.00 | 0.10 | 0.00 | 0.00 |
| Kazan-3B-28 | 0.25 | 0.00 | 0.00 | 0.00 | 0.00 | 0.00 | 0.00 | 0.12 | 0.00 | 0.00 | 0.00 |
| Fusobacteria | 0.00 | 0.00 | 0.00 | 0.00 | 0.00 | 0.00 | 0.00 | 0.03 | 0.00 | 0.14 | 0.00 |
| AD3 | 0.00 | 0.00 | 0.00 | 0.00 | 0.00 | 0.00 | 0.00 | 0.00 | 0.20 | 0.00 | 0.00 |
| WS6 | **1.00** | **1.14** | 0.71 | 0.53 | 0.00 | 0.00 | 0.00 | 0.26 | 0.00 | 0.00 | 0.00 |
| OP11 | **1.05** | **2.57** | **2.10** | **11.23** | **5.63** | 0.00 | 0.00 | 0.26 | 0.00 | 0.00 | 0.00 |
| FCPU426 | 0.00 | 0.03 | 0.00 | 0.00 | 0.00 | 0.00 | 0.00 | 0.00 | 0.00 | 0.00 | 0.31 |
| Chlorobi | 0.25 | 0.63 | **1.68** | 0.36 | 0.12 | 0.00 | 0.00 | 0.03 | 0.31 | 0.00 | 0.00 |
| Gemmatimonadetes | 0.00 | 0.00 | 0.00 | 0.00 | 0.00 | 0.08 | 0.00 | 0.06 | 0.20 | 0.00 | 0.08 |
| NC10 | 0.00 | 0.00 | 0.00 | 0.00 | 0.25 | 0.00 | 0.00 | 0.00 | 0.00 | 0.00 | 0.38 |
| Spirochaetes | 0.21 | 0.70 | **1.03** | 0.07 | 0.12 | 0.08 | 0.08 | 0.00 | 0.00 | 0.00 | 0.31 |
| WWE1 | **10.38** | **1.01** | **2.25** | 0.07 | 0.00 | 0.00 | 0.04 | 0.00 | 0.31 | 0.00 | 0.08 |
| Fibrobacteres | 0.00 | 0.00 | 0.00 | 0.00 | 0.00 | 0.00 | 0.00 | 0.00 | 0.00 | 0.00 | 0.54 |
| OP3 | 0.04 | **2.03** | **1.96** | 0.13 | **4.33** | 0.00 | 0.00 | 0.00 | 0.00 | 0.00 | 0.54 |
| TM6 | 0.21 | 0.32 | 0.61 | 0.50 | 0.06 | 0.25 | 0.00 | **0.59** | 0.00 | 0.00 | 0.00 |
| Chlamydiae | 0.13 | 0.00 | 0.18 | 0.13 | 0.80 | **1.01** | 0.00 | 0.67 | 0.10 | 0.00 | 0.23 |
| Chloroflexi | 0.59 | 0.22 | 0.25 | 0.43 | 0.31 | 0.17 | 0.04 | 0.32 | 0.20 | 0.14 | 0.38 |
| Tenericutes | **1.84** | 0.19 | 0.07 | 0.03 | 0.00 | 0.00 | 0.21 | 0.47 | 0.41 | 0.00 | 0.23 |
| GN02 | 0.17 | **6.49** | **3.89** | **4.50** | **8.61** | 0.25 | 0.00 | **1.14** | 0.20 | 0.14 | 0.08 |
| SR1 | 0.00 | 0.10 | 0.07 | 0.43 | 0.25 | 0.17 | 0.00 | **2.40** | 0.00 | 0.00 | 0.00 |
| Acidobacteria | 0.00 | 0.19 | 0.36 | 0.33 | 0.19 | 0.93 | 0.00 | 0.09 | **1.23** | 0.54 | **1.68** |
| Nitrospirae | 0.00 | 0.16 | 0.21 | 0.23 | 0.12 | 0.42 | 0.00 | 0.03 | 0.00 | **3.54** | 0.54 |
| Planctomycetes | 0.17 | 0.38 | **1.61** | 0.23 | 0.87 | **2.02** | 0.00 | **1.05** | **2.04** | 0.68 | **1.53** |
| Bacteria_Other | **4.35** | **3.14** | **4.89** | **9.30** | **6.75** | **1.18** | 0.50 | 0.91 | **2.25** | 0.68 | **1.07** |
| Elusimicrobia | 0.00 | 0.51 | 0.54 | 0.07 | **1.05** | 0.17 | 0.00 | 0.03 | **1.23** | 0.95 | **6.05** |
| Actinobacteria | 0.59 | 0.48 | 0.57 | **2.35** | 0.74 | **3.62** | **1.30** | **1.41** | **5.31** | **1.77** | **2.91** |
| TM7 | 0.08 | 0.48 | 0.39 | **4.17** | **3.22** | **1.01** | 0.46 | **1.93** | **4.49** | **7.36** | 0.38 |
| Cyanobacteria | 0.17 | 0.32 | 0.43 | 0.66 | 0.19 | 0.42 | 0.08 | **18.13** | 0.72 | 0.14 | 0.38 |
| OD1 | **9.62** | **28.35** | **36.71** | **30.50** | **53.50** | 0.59 | 0.04 | **18.39** | 0.72 | 0.82 | **4.82** |
| Firmicutes | **35.61** | **2.09** | **2.50** | **13.91** | **5.26** | **1.51** | **36.12** | **3.75** | **4.39** | 0.27 | **2.91** |
| Verrucomicrobia | 0.54 | 0.38 | **1.07** | **1.72** | 0.56 | **18.42** | 0.42 | **3.08** | **24.92** | **11.72** | **15.77** |
| Bacteroidetes | **15.90** | **12.48** | **14.77** | **1.85** | **3.10** | **14.13** | **36.92** | **11.57** | **13.79** | **10.63** | **22.89** |
