# Supplementary material for: Impacts of leachates from livestock carcass burial and manure heap sites on groundwater geochemistry and microbial community structure
Source: PLoS One. 2017 Aug 3;12(8):e0182579. doi: 10.1371/journal.pone.0182579 (PMC5542392; doi:10.1371/journal.pone.0182579)
Supplement: S3 Table — (DOCX) [file pone.0182579.s005.docx]

S3 Table. Classification and relative abundance of Bacteria (genus level) at the livestock carcasses burial and livestock manure heap sites.

| **#OTU ID** | **Livestock carcass burial site** | | | | | | **Livestock manure heap site** | | | | |
| --- | --- | --- | --- | --- | --- | --- | --- | --- | --- | --- | --- |
|  | **IH** | **IA4** | **IA3** | **IA1** | **IB3** | **ID** | **YH** | **YG** | **YB1** | **YC2** | **YC3** |
| p_OD1 | **5.98** | **12.99** | **21.33** | **7.02** | **15.48** | 0.00 | 0.00 | **1.23** | 0.00 | 0.00 | 0.46 |
| p_OD1;c_ZB2;o_unclassified;f_unclassified;g_unclassified | 0.42 | **6.53** | **7.71** | **8.94** | **15.79** | 0.42 | 0.04 | **7.06** | 0.20 | 0.54 | **2.53** |
| p_OD1;c_ABY1;o_unclassified;f_unclassified;g_unclassified | **1.80** | **6.65** | **5.03** | **9.93** | **10.71** | 0.00 | 0.00 | **1.05** | 0.20 | 0.14 | 0.15 |
| p_OD1;c_SM2F11;o_unclassified;f_unclassified;g_unclassified | 0.04 | 0.60 | 0.71 | **2.15** | **5.39** | 0.17 | 0.00 | **1.03** | 0.31 | 0.14 | 0.23 |
| p_OD1;c_Mb-NB09;o_unclassified;f_unclassified;g_unclassified | 0.00 | 0.00 | 0.14 | 0.93 | **2.04** | 0.00 | 0.00 | **7.97** | 0.00 | 0.00 | **1.45** |
| p_OD1;Other;Other;Other;Other | **1.38** | **1.58** | **1.78** | **1.52** | **4.09** | 0.00 | 0.00 | 0.06 | 0.00 | 0.00 | 0.00 |
| p_OP3;c_koll11;o_GIF10;f_unclassified;g_unclassified | 0.04 | **1.46** | **1.50** | 0.07 | **4.21** | 0.00 | 0.00 | 0.00 | 0.00 | 0.00 | 0.00 |
| p_OP11;c_OP11-4;o_unclassified;f_unclassified;g_unclassified | 0.00 | 0.38 | 0.32 | **5.79** | **3.53** | 0.00 | 0.00 | 0.03 | 0.00 | 0.00 | 0.00 |
| p_OP11;c_OP11-3;o_unclassified;f_unclassified;g_unclassified | 0.46 | **1.68** | **1.36** | **2.58** | 0.31 | 0.00 | 0.00 | 0.18 | 0.00 | 0.00 | 0.00 |
| p_OP11;c_WCHB1-64;o_K2-4-19;f_unclassified;g_unclassified | 0.00 | 0.38 | 0.36 | **2.52** | 0.99 | 0.00 | 0.00 | 0.03 | 0.00 | 0.00 | 0.00 |
| p_GN02;c_BD1-5;o_unclassified;f_unclassified;g_unclassified | 0.04 | **3.20** | 0.82 | 0.20 | 0.06 | 0.25 | 0.00 | 0.70 | 0.10 | 0.14 | 0.08 |
| p_GN02;c_3BR-5F;o_unclassified;f_unclassified;g_unclassified | 0.04 | 0.16 | 0.50 | **2.42** | **1.73** | 0.00 | 0.00 | 0.29 | 0.00 | 0.00 | 0.00 |
| p_GN02;c_GKS2-174;o_unclassified;f_unclassified;g_unclassified | 0.08 | 2.91 | **2.35** | **1.13** | **5.94** | 0.00 | 0.00 | 0.15 | 0.00 | 0.00 | 0.00 |
| p_SR1 | 0.00 | 0.10 | 0.07 | 0.43 | 0.25 | 0.17 | 0.00 | **2.40** | 0.00 | 0.00 | 0.00 |
| p_TM7;c_TM7-1;o_unclassified;f_unclassified;g_unclassified | 0.00 | 0.19 | 0.36 | **3.84** | **2.60** | 0.34 | 0.04 | **1.20** | **2.35** | 0.00 | 0.23 |
| p_TM7;c_TM7-3;o_unclassified;f_unclassified;g_unclassified | 0.00 | 0.00 | 0.00 | 0.00 | 0.00 | 0.00 | 0.04 | 0.00 | **1.43** | **6.40** | 0.00 |
| p_WWE1;c_[Cloacamonae];o_[Cloacamonales];f_[Cloacamonaceae];g_W22 | **8.08** | 0.41 | 0.64 | 0.00 | 0.00 | 0.00 | 0.04 | 0.00 | 0.20 | 0.00 | 0.08 |
| p_WWE1;c_[Cloacamonae];o_[Cloacamonales];f_[Cloacamonaceae];g_ | **1.13** | 0.57 | **1.53** | 0.07 | 0.00 | 0.00 | 0.00 | 0.00 | 0.00 | 0.00 | 0.00 |
| p_WS6;c_SC72;o_WCHB1-15;f_unclassified;g_unclassified | 0.42 | **1.05** | 0.64 | 0.46 | 0.00 | 0.00 | 0.00 | 0.00 | 0.00 | 0.00 | 0.00 |
| Other;Other;Other;Other;Other | **4.35** | **3.14** | **4.89** | **9.30** | **6.75** | **1.18** | 0.50 | 0.91 | **2.25** | 0.68 | **1.07** |
| p_Elusimicrobia;c_Elusimicrobia;o_Elusimicrobiales;f_unclassified;g_unclassified | 0.00 | 0.38 | 0.32 | 0.07 | 0.00 | 0.17 | 0.00 | 0.00 | **1.02** | 0.95 | **5.97** |
| p_Planctomycetes;c_Planctomycetia;o_Pirellulales;f_Pirellulaceae;g_ | 0.17 | 0.03 | 0.14 | 0.03 | 0.00 | **1.01** | 0.00 | 0.67 | 0.92 | 0.54 | 0.31 |
| p_Cyanobacteria;c_Chloroplast;o_Stramenopiles;f_unclassified;g_unclassified | 0.00 | 0.00 | 0.00 | 0.00 | 0.00 | 0.17 | 0.00 | **6.74** | 0.00 | 0.14 | 0.08 |
| p_Cyanobacteria;c_Chloroplast;o_Chlorophyta;f_Chlamydomonadaceae;g_ | 0.00 | 0.00 | 0.00 | 0.00 | 0.00 | 0.00 | 0.00 | **2.55** | 0.00 | 0.00 | 0.00 |
| p_Nitrospirae;c_Nitrospira;o_Nitrospirales;f_Nitrospiraceae;g_Nitrospira | 0.00 | 0.00 | 0.04 | 0.00 | 0.00 | 0.42 | 0.00 | 0.03 | 0.00 | **3.54** | 0.31 |
| p_Verrucomicrobia;c_[Methylacidiphilae];o_Methylacidiphilales;f_Methylacidiphilaceae;g_Candidatus Methylacidiphilum | 0.54 | 0.06 | 0.18 | **1.42** | 0.50 | **17.75** | 0.42 | **1.64** | **23.70** | **7.90** | **14.55** |
| p_Verrucomicrobia;c_Opitutae;o_Opitutales;f_Opitutaceae;g_ | 0.00 | 0.00 | 0.00 | 0.00 | 0.06 | 0.17 | 0.00 | 0.00 | 0.00 | **2.86** | 0.38 |
| p_Synergistetes;c_Synergistia;o_Synergistales;f_Synergistaceae;g_vadinCA02 | **1.67** | 0.54 | 0.54 | 0.33 | 0.00 | 0.00 | 0.00 | 0.00 | 0.00 | 0.00 | 0.00 |
| p_Actinobacteria;c_Acidimicrobiia;o_Acidimicrobiales;f_C111;g_ | 0.00 | 0.03 | 0.00 | 0.17 | 0.00 | 0.76 | 0.00 | 0.26 | **1.02** | 0.00 | 0.77 |
| p_Actinobacteria;c_Actinobacteria;o_Actinomycetales;Other;Other | 0.04 | 0.00 | 0.04 | 0.13 | 0.00 | 0.25 | 0.17 | 0.32 | 0.82 | 0.41 | 0.31 |
| p_Firmicutes;c_Clostridia;o_Clostridiales;f_Clostridiaceae;g_Clostridium | **2.38** | 0.06 | 0.14 | **1.72** | 0.00 | 0.00 | **1.51** | 0.53 | 0.00 | 0.00 | 0.15 |
| p_Firmicutes;c_Clostridia;o_Clostridiales;f_Ruminococcaceae;g_ | **4.06** | 0.10 | 0.04 | 0.10 | 0.00 | 0.08 | 0.80 | 0.12 | 0.10 | 0.00 | 0.08 |
| p_Firmicutes;c_Clostridia;o_Clostridiales;f_Ruminococcaceae;Other | 0.67 | 0.06 | 0.11 | 0.10 | 0.00 | 0.17 | 0.17 | 0.06 | 0.51 | 0.00 | 0.38 |
| p_Firmicutes;c_Clostridia;o_Clostridiales;f_unclassified;g_unclassified | **1.63** | 0.00 | 0.11 | 0.03 | 0.00 | 0.00 | **1.38** | 0.15 | 0.00 | 0.00 | 0.00 |
| p_Firmicutes;c_Clostridia;o_Clostridiales;f_Syntrophomonadaceae;g_Syntrophomonas | **15.82** | 0.22 | 0.18 | 0.56 | 0.06 | 0.00 | 0.00 | 0.03 | 0.41 | 0.00 | 0.31 |
| p_Firmicutes;c_Clostridia;o_Clostridiales;f_Peptococcaceae;g_Desulfosporosinus | 0.08 | 0.92 | 0.78 | **5.73** | **3.22** | 0.08 | 0.00 | 0.00 | 0.20 | 0.00 | 0.00 |
| p_Firmicutes;c_Clostridia;o_Clostridiales;Other;Other | **2.13** | 0.03 | 0.14 | 0.60 | 0.06 | 0.00 | 0.33 | 0.09 | 0.20 | 0.00 | 0.08 |
| p_Firmicutes;c_Bacilli;o_Bacillales;f_Planococcaceae;g_Solibacillus | 0.00 | 0.00 | 0.00 | 0.00 | 0.00 | 0.00 | **5.53** | 0.00 | 0.00 | 0.00 | 0.00 |
| p_Firmicutes;c_Bacilli;o_Lactobacillales;f_Aerococcaceae;g_ | 0.00 | 0.00 | 0.00 | 0.00 | 0.00 | 0.00 | **3.26** | 0.12 | 0.00 | 0.00 | 0.00 |
| p_Firmicutes;Other;Other;Other;Other | 0.50 | 0.16 | 0.14 | 0.83 | 0.43 | 0.00 | 0.25 | 0.06 | 0.10 | 0.00 | 0.00 |
| p_Bacteroidetes;c_[Saprospirae];o_[Saprospirales];f_Chitinophagaceae;Other | 0.08 | 0.03 | 0.04 | 0.03 | 0.00 | **1.26** | 0.00 | 0.09 | **1.23** | 0.27 | 0.92 |
| p_Bacteroidetes;c_[Saprospirae];o_[Saprospirales];f_Chitinophagaceae;g_Sediminibacterium | 0.13 | 0.03 | 0.07 | 0.00 | 0.06 | 0.67 | 0.00 | 0.53 | 0.20 | **1.63** | **2.83** |
| p_Bacteroidetes;c_[Saprospirae];o_[Saprospirales];f_Chitinophagaceae;g_ | 0.08 | 0.16 | 0.14 | 0.13 | 0.06 | **1.09** | 0.08 | 0.21 | **1.02** | 0.27 | 0.46 |
| p_Bacteroidetes;Other;Other;Other;Other | 0.63 | 0.63 | 0.61 | 0.03 | 0.19 | 0.42 | 0.42 | 0.06 | 0.51 | 0.14 | 0.23 |
| p_Bacteroidetes;c_Flavobacteriia;o_Flavobacteriales;f_Flavobacteriaceae;g_ | 0.00 | 0.00 | 0.00 | 0.00 | 0.00 | 0.00 | **9.04** | 0.12 | 0.10 | 0.00 | 0.08 |
| p_Bacteroidetes;c_Flavobacteriia;o_Flavobacteriales;f_Flavobacteriaceae;g_Flavobacterium | 0.04 | 0.06 | 0.00 | 0.03 | 0.12 | 0.67 | **13.94** | **5.89** | 1.12 | 0.68 | 0.69 |
| p_Bacteroidetes;c_Bacteroidia;o_Bacteroidales;f_Bacteroidaceae;g_ | 0.00 | **4.18** | **4.78** | 0.20 | 0.00 | 0.00 | 0.17 | 0.00 | 0.00 | 0.00 | 0.00 |
| p_Bacteroidetes;c_Bacteroidia;o_Bacteroidales;f_Bacteroidaceae;g_Bacteroides | 0.71 | 0.19 | 0.04 | 0.13 | 0.12 | 0.59 | 0.08 | 0.15 | 0.92 | 0.41 | 0.61 |
| p_Bacteroidetes;c_Bacteroidia;o_Bacteroidales;f_Porphyromonadaceae;g_ | **6.23** | 0.16 | 0.21 | 0.03 | 0.00 | 0.00 | **5.78** | 0.67 | 0.00 | 0.00 | 0.00 |
| p_Bacteroidetes;c_Bacteroidia;o_Bacteroidales;f_unclassified;g_unclassified | **5.69** | **5.51** | **6.89** | 0.70 | **1.42** | 0.25 | 0.54 | 0.18 | 0.31 | 0.00 | 0.15 |
| p_Bacteroidetes;c_Sphingobacteriia;o_Sphingobacteriales;f_Sphingobacteriaceae;g_ | 0.13 | 0.00 | 0.00 | 0.00 | 0.43 | 0.42 | 0.08 | 0.06 | 0.61 | **4.50** | 0.92 |
| p_Bacteroidetes;c_Sphingobacteriia;o_Sphingobacteriales;f_unclassified;g_unclassified | 0.04 | 0.63 | 0.50 | 0.26 | 0.50 | **5.72** | 0.00 | 0.09 | **3.27** | 0.00 | **12.40** |
| p_Proteobacteria;Other;Other;Other;Other | 0.04 | 0.51 | 0.39 | 0.89 | 0.31 | 0.34 | 0.00 | 0.26 | 0.20 | 0.27 | 0.15 |
| p_Proteobacteria;c_Alphaproteobacteria;o_Caulobacterales;f_Caulobacteraceae;g_ | 0.00 | 0.03 | 0.00 | 0.00 | 0.00 | 0.76 | 0.04 | 0.03 | 0.00 | **1.36** | 1.15 |
| p_Proteobacteria;c_Alphaproteobacteria;o_Rhodospirillales;f_Rhodospirillaceae;g_Magnetospirillum | 0.00 | 0.16 | 0.00 | 0.00 | 0.00 | 0.00 | 0.00 | 0.00 | 0.20 | **2.18** | 0.00 |
| p_Proteobacteria;c_Alphaproteobacteria;o_Rhodobacterales;f_Rhodobacteraceae;Other | 0.00 | 0.00 | 0.00 | 0.00 | 0.06 | 0.34 | **1.42** | 0.62 | 0.31 | 0.14 | 0.15 |
| p_Proteobacteria;c_Alphaproteobacteria;o_Rhodobacterales;f_Rhodobacteraceae;g_Rhodobacter | 0.00 | 0.00 | 0.00 | 0.00 | 0.00 | 0.00 | 0.29 | **11.10** | 0.00 | 0.14 | 0.00 |
| p_Proteobacteria;c_Alphaproteobacteria;o_Rhodobacterales;f_Rhodobacteraceae;g__ | 0.00 | 0.00 | 0.00 | 0.03 | 0.00 | **1.43** | 0.08 | 0.09 | **3.17** | 0.27 | **1.53** |
| p_Proteobacteria;c_Alphaproteobacteria;o_Sphingomonadales;f_Sphingomonadaceae;g_Sphingomonas | 0.04 | 0.03 | 0.00 | 0.00 | 0.00 | **1.77** | 0.00 | 0.06 | 0.41 | **1.63** | 0.38 |
| p_Proteobacteria;c_Alphaproteobacteria;o_Sphingomonadales;f_Erythrobacteraceae;g_Erythromicrobium | 0.00 | 0.00 | 0.00 | 0.00 | 0.00 | 0.17 | 0.00 | **2.84** | 0.00 | 0.00 | 0.00 |
| p_Proteobacteria;c_Alphaproteobacteria;o_Sphingomonadales;f_Sphingomonadaceae;g_Novosphingobium | 0.00 | 0.00 | 0.00 | 0.00 | 0.00 | 0.42 | 0.08 | 0.41 | 0.41 | **22.48** | **2.76** |
| p_Proteobacteria;c_Alphaproteobacteria;o_Ellin329;f_unclassified;g_unclassified | 0.00 | 0.00 | 0.00 | 0.00 | 0.00 | 0.00 | 0.00 | 0.00 | 0.31 | **1.63** | 0.38 |
| p_Proteobacteria;c_Alphaproteobacteria;o_Rhizobiales;Other;Other | **1.09** | 0.00 | 0.04 | 0.10 | 0.00 | 0.34 | 0.04 | 0.53 | 0.10 | 0.14 | 0.31 |
| p_Proteobacteria;c_Alphaproteobacteria;o_Rhizobiales;f_Methylocystaceae;g_Methylosinus | **4.14** | 0.06 | 0.11 | 0.33 | 0.00 | 0.00 | 0.00 | 0.09 | 0.00 | 0.00 | 0.00 |
| p_Proteobacteria;c_Alphaproteobacteria;Other;Other;Other | 0.13 | 0.03 | 0.18 | 0.13 | 0.12 | 0.25 | 0.00 | 0.56 | 0.31 | **1.77** | 0.46 |
| p_Proteobacteria;c_Betaproteobacteria;Other;Other;Other | 0.29 | **1.14** | **1.78** | **1.06** | 0.00 | **7.65** | 0.04 | 0.26 | **1.33** | 0.82 | 0.31 |
| p_Proteobacteria;c_Betaproteobacteria;o_Gallionellales;f_Gallionellaceae;g_Gallionella | 0.17 | 0.63 | **1.18** | 0.40 | 0.00 | **15.56** | 0.00 | 0.00 | **2.86** | 0.00 | 0.00 |
| p_Proteobacteria;c_Betaproteobacteria;o_Burkholderiales;f_Comamonadaceae;g_Acidovorax | 0.08 | 0.00 | 0.07 | 0.07 | 0.00 | 0.00 | 0.00 | **1.61** | 0.10 | 0.27 | 0.23 |
| p_Proteobacteria;c_Betaproteobacteria;o_Burkholderiales;f_Oxalobacteraceae;g_Cupriavidus | 0.00 | 0.00 | 0.00 | 0.03 | 0.00 | 0.76 | 0.00 | 0.09 | **1.23** | 0.14 | 0.46 |
| p_Proteobacteria;c_Betaproteobacteria;o_Burkholderiales;f_Comamonadaceae;g_Rhodoferax | 0.13 | 0.25 | 0.29 | 0.50 | 0.00 | 0.00 | 0.00 | **1.23** | 0.72 | **1.36** | 0.38 |
| p_Proteobacteria;c_Betaproteobacteria;o_Burkholderiales;f_Comamonadaceae;Other | 0.54 | 0.10 | 0.21 | 0.23 | 0.00 | 0.34 | 0.67 | 0.70 | 0.51 | 0.41 | 0.31 |
| p_Proteobacteria;c_Betaproteobacteria;o_Methylophilales;f_unclassified;g_unclassified | 0.00 | **1.33** | **2.53** | **4.04** | 0.00 | 0.00 | 0.00 | 0.00 | 0.41 | 0.00 | 0.00 |
| p_Proteobacteria;c_Gammaproteobacteria;o_Methylococcales;f_Crenotrichaceae;g_Crenothrix | 0.79 | 0.86 | 0.82 | 0.03 | 0.12 | 0.00 | 0.00 | 0.00 | 0.72 | 0.00 | 0.00 |
| p_Proteobacteria;c_Gammaproteobacteria;o_Pseudomonadales;f_Pseudomonadaceae;g_Pseudomonas | 0.00 | 0.00 | 0.00 | 0.07 | 0.00 | 0.00 | 0.67 | 0.21 | 0.00 | **1.36** | 0.31 |
| p_Proteobacteria;c_Gammaproteobacteria;o_Pseudomonadales;f_Moraxellaceae;g_Acinetobacter | 0.00 | 0.00 | 0.00 | 0.00 | 0.00 | 0.00 | **12.56** | 0.18 | 0.00 | 0.00 | 0.15 |
| p_Proteobacteria;c_Gammaproteobacteria;o_Pseudomonadales;f_Moraxellaceae;g_Perlucidibaca | 0.00 | 0.00 | 0.00 | 0.00 | 0.00 | 0.00 | 0.00 | 0.03 | 3.58 | 0.27 | **4.13** |
| p_Proteobacteria;c_Gammaproteobacteria;o_Enterobacteriales;f_Enterobacteriaceae;Other | 0.08 | 0.10 | 0.07 | 0.03 | 0.06 | 0.84 | 0.04 | 0.23 | 0.82 | 0.82 | 0.84 |
| p_Proteobacteria;c_Gammaproteobacteria;o_Vibrionales;f_Vibrionaceae;g_ | 0.04 | 0.00 | 0.04 | 0.07 | 0.06 | **1.43** | 0.00 | 0.15 | **1.43** | 0.27 | 0.84 |
| p_Proteobacteria;c_Gammaproteobacteria;o_Xanthomonadales;f_Xanthomonadaceae;g_Rhodanobacter | 0.00 | 0.00 | 0.00 | 0.03 | 0.00 | 0.00 | 0.00 | 0.00 | 0.20 | **7.49** | 0.08 |
| p_Proteobacteria;c_Gammaproteobacteria;o_unclassified;f_unclassified;g_unclassified | 0.00 | 0.00 | 0.00 | 0.00 | 0.00 | **5.47** | 0.00 | 0.00 | 0.00 | 0.00 | 0.08 |
| p_Proteobacteria;c_Gammaproteobacteria;Other;Other;Other | 0.04 | 0.10 | 0.04 | 0.53 | 0.06 | 0.76 | 0.33 | 0.06 | 0.41 | 0.41 | 1.00 |
| p_Proteobacteria;c_Epsilonproteobacteria;o_Campylobacterales;f_Helicobacteraceae;g_Sulfurimonas | 0.00 | **21.79** | **4.35** | 0.17 | 0.00 | 0.00 | 0.00 | 0.00 | 0.00 | 0.00 | 0.00 |
