# Supplementary material for: Impacts of leachates from livestock carcass burial and manure heap sites on groundwater geochemistry and microbial community structure
Source: PLoS One. 2017 Aug 3;12(8):e0182579. doi: 10.1371/journal.pone.0182579 (PMC5542392; doi:10.1371/journal.pone.0182579)
Supplement: S4 Table — (DOCX) [file pone.0182579.s006.docx]

S4 Table. Classification and relative abundance of Archaea (genus level) at the livestock carcass burial and livestock manure heap sites.
