# Supplementary material for: Impacts of leachates from livestock carcass burial and manure heap sites on groundwater geochemistry and microbial community structure
Source: PLoS One. 2017 Aug 3;12(8):e0182579. doi: 10.1371/journal.pone.0182579 (PMC5542392; doi:10.1371/journal.pone.0182579)
Supplement: S5 Table — (DOCX) [file pone.0182579.s007.docx]

Table S5. NMDS data Non-metric multidimensional scaling (NMDS) data of environmental variables and microbial community compositions at the phylum level in water samples collected from the livestock burial site (A) and both the livestock burial and manure heap sites (B).

| **A** NMDS1 NMDS2 r2 Pr(>r) |
| --- |
| Distance.from.source 0.98909 0.14730 0.9459 0.05000 * |
| Sampling.depth..m. 0.99846 0.05553 0.8480 0.10000 . |
| Temp. -0.96260 -0.27093 0.9394 0.04306 * |
| pH 0.46779 -0.88384 0.9331 0.02222 * |
| Eh 0.37132 0.92851 0.9179 0.03611 * |
| EC -0.31643 -0.94862 0.6610 0.17361 |
| DO 0.97420 0.22567 0.9672 0.01250 * |
| Turbidity -0.26767 -0.96351 0.5755 0.19444 |
| Total.colony.count -0.27742 -0.96075 0.5852 0.19444 |
| Na. -0.37194 -0.92826 0.6542 0.19722 |
| Cl. -0.27629 -0.96107 0.5863 0.17500 |
| SO42. -0.27441 -0.96161 0.5861 0.18889 |
| NO3. -0.26680 -0.96375 0.5734 0.26944 |
| HCO3. -0.27253 -0.96215 0.5870 0.23056 |

Significance codes: ‘***’ 0.001 ‘**’ 0.01 ‘*’ 0.05 ‘.’ 0.1 ‘ ’ 1

Permutation: free

Number of permutations: 719

| **B**  NMDS1 NMDS2 r2 Pr(>r) |
| --- |
| Sampling.depth.m. 0.75470 -0.65606 0.2122 0.4146 |
| Temp. -0.97966 0.20067 0.1782 0.5061 |
| pH -0.08403 0.99646 0.4529 0.1025 |
| Eh 0.66772 -0.74442 0.7651 0.0106 * |
| EC -0.40572 0.91400 0.6667 0.0543 . |
| DO 0.72788 0.68570 0.6222 0.0348 * |
| Turbidity -0.39221 0.91987 0.6787 0.0216 * |
| Total.colony.count -0.09481 0.99550 0.5614 0.0797 . |
| Na. 0.40537 0.91415 0.2396 0.3805 |
| Cl. -0.34667 0.93799 0.7074 0.0223 * |
| SO42. -0.35560 0.93464 0.7349 0.0107 * |
| NO3. -0.35785 0.93378 0.7285 0.0073 ** |
| HCO3. -0.40802 0.91297 0.6780 0.0416 * |

Significance codes: ‘***’ 0.001 ‘**’ 0.01 ‘*’ 0.05 ‘.’ 0.1 ‘ ’ 1

Permutation: free

Number of permutations: 9999
